# Long-term trends in DM and dementia-related mortality among middle-aged and elderly adults in the United States, 1999–2023: a nationwide population-based study

**DOI:** 10.1186/s12889-026-27257-9

**Published:** 2026-04-14

**Authors:** Wenyan Xu, Qiuling Xing, Min Ding

**Affiliations:** NHC Key Lab of Hormones and Development and Tianjin Key Lab of Metabolic Diseases, Tianjin Medical University Chu Hsien-I Memorial Hospital & Institute of Endocrinology, No.6 North Huanrui Rd, Beichen District, Tianjin, 300134 China

**Keywords:** Diabetes mellitus, Dementia, CDC WONDER, Mortality, Health disparities

## Abstract

**Background:**

Diabetes mellitus (DM) and dementia frequently coexist in middle-aged and elderly people, jointly amplifying mortality risk. Clarifying long-term mortality patterns is essential for informing prevention and resource allocation.

**Methods:**

We conducted a nationwide, population-based analysis using CDC WONDER Multiple Cause of Death data (1999–2023). Population mortality rates were calculated among decedents with both DM (ICD-10 E10-E14) and dementia (F01, F03, G30) recorded on the death certificate. Age-adjusted mortality rates (AAMRs) were standardized to the 2000 U.S. population. Temporal trends were evaluated using Joinpoint regression and ARIMA models stratified by sex, race/ethnicity, region, and urbanization.

**Results:**

A total of 749,517 DM and dementia-related deaths occurred during 1999–2023, with AAMR rising from 12.05 to 30.95 per 100,000 (AAPC = 3.48%, *P* < 0.05). Males and NH Black individuals consistently demonstrated the highest mortality. Rural areas showed persistently elevated AAMRs, alongside marked interstate variation.

**Conclusion:**

DM and Dementia-related mortality among U.S. adults aged ≥ 45 years increased nearly 2.57-fold over the past two decades, with enduring sex, racial, and geographic disparities. Strengthening equitable, community-based preventive strategies and integrated metabolic–cognitive care is urgently needed.

**Supplementary Information:**

The online version contains supplementary material available at 10.1186/s12889-026-27257-9.

## Introduction

Diabetes mellitus (DM) and dementia are among the most prevalent and disabling chronic diseases worldwide, posing an escalating challenge to global public health. Over the past two decades, the prevalence of diabetes has continued to rise, driven largely by population aging, sedentary lifestyles, and dietary transitions [1]. In the United States, diabetes affects more than 11.6% of adults (3840 million people), contributing substantially to morbidity and premature mortality each year [2, 3]. Meanwhile, dementia has become one of the leading causes of death and disability among adults. The latest WHO statistics show that there are currently 55.2 million people living with dementia worldwide, with 65% of deaths attributed to dementia-related diseases [4]. Globally, approximately 55 million people are living with dementia, and this number is projected to rise to 152.8 million cases by 2050 [5]. This trend not only reflects the prolongation of human lifespan but also highlights the lack of effective treatment options.

Alarmingly, both diabetes and dementia are showing a shifting age pattern, with a growing proportion of new cases now occurring in middle-aged adults. Large-scale cohort studies reveal that individuals diagnosed with diabetes in middle age have a cumulative dementia incidence of up to 25.2% [7] by age 80—more than double the 11.5% seen in those without diabetes [6]. For every 5 years earlier that diabetes onset occurs, the subsequent risk of dementia increases by 24%. Overall, people with diabetes develop dementia an average of 2.2 years earlier, with vascular dementia occurring up to 3 years earlier [8]. This shift is closely linked to the rising prevalence of obesity, hypertension, and insulin resistance in midlife, resulting in an increasing number of new cases emerging before age 65, and even before age 55.

Importantly, both conditions share several modifiable risk factors—including vascular dysfunction, obesity, and physical inactivity—implying that they are, at least in part, preventable or reversible through early detection and intervention. It is estimated that up to 40% of dementia cases and a substantial proportion of diabetes-related complications could be delayed or prevented by controlling metabolic and vascular risks during midlife [9]. One of the most severe consequences of this co-morbidity is the markedly elevated mortality risk. Analyses show that the age-adjusted mortality rate for Alzheimer’s disease among individuals with type 2 diabetes rose dramatically from 4.12 per 100,000 in 1999 to 11.65 per 100,000 in 2019 [10]. In postmenopausal women, type 2 diabetes is associated with a cause-specific hazard ratio of 2.94 for dementia-related death, significantly higher than the 2.65 observed for non-dementia deaths [11].

Despite these well-established associations, critical evidence gaps persist in the literature. Few studies have systematically quantified the mortality trajectories and age-specific transition probabilities of diabetes-dementia co-morbidity among adults aged 45 years and older. Furthermore, existing research predominantly examines type 2 diabetes or Alzheimer’s disease in isolation, whereas the dynamic evolution of the mortality burden encompassing all types of diabetes and all-cause dementia remains inadequately described. Our study aims to characterize racial disparities in dementia and DM-related mortality among U.S. adults aged 45 and older from 1999 to 2023, while accounting for temporal trends and variations by sex, geographic region, and urban-rural residence. These findings are expected to elucidate underlying mechanisms and provide a robust evidence base for the development of future targeted interventions.

## Methods

### Study design and population

We performed a population-based, retrospective analysis using data from the Centers for Disease Control and Prevention’ s Wide-ranging Online Data for Epidemiologic Research (CDC WONDER) platform. Mortality data were extracted from the Multiple Cause of Death (MCOD) files covering the years 1999–2023. This dataset compiles information from death certificates issued across all U.S. states, including demographic variables, underlying causes, and contributing conditions listed on each record.

Our study investigated dementia and DM-related deaths in individuals aged 45 years and older, reflecting the age range where dementia becomes more clinically relevant and its prevalence rises substantially with advancing age^9^. Cases were identified based on the population mortality rate of decedents with both diabetes and dementia recorded on the death certificate. According to the International Classification of Diseases, 10th Revision (ICD-10), diabetes was defined by codes E10-E14, and dementia was defined by F01 (vascular dementia), F03 (unspecified dementia), and G30 (Alzheimer’s disease) [12, 13]. Additionally, we separately extracted data on deaths in which dementia or diabetes was recorded as the underlying cause of death, with the other condition listed as a contributing cause.

Because the CDC WONDER database contains only publicly available, de-identified data, this study was exempt from institutional review board approval and informed consent. All procedures followed the Strengthening the Reporting of Observational Studies in Epidemiology (STROBE) guidelines for observational research.

### Data abstraction

Demographic and contextual variables were abstracted from the CDC WONDER Multiple Cause of Death database to ensure consistency in data classification and comparability across years. Information extracted from death certificates included age, sex, race or ethnicity, geographic region, and place of death, which were categorized according to standardized definitions established by the U.S. National Center for Health Statistics (NCHS) and the U.S. Census Bureau. The place of death was categorized into: inpatient hospital, emergency department, outpatient or clinic, home, hospice, nursing home or long-term care facility, and other or unknown locations. Race and ethnicity were classified as Hispanic (Latino) and non-Hispanic (NH) groups. The NH category included NH White, NH Black or African American, and NH Other (including Asian, Pacific Islander, American Indian, and Alaska Native populations). Due to incomplete or suppressed data for smaller race categories in certain years, these subgroups were aggregated as NH Others.

Age groups were defined as 45–54, 55–64, 65–74, 75–84, and ≥ 85 years, in line with the stratification used in the results section. Geographic classification included both Census regions and urban–rural status. The four Census regions—Northeast, Midwest, South, and West—were defined according to the U.S. Census Bureau standards. The urban–rural classification followed the 2013 NCHS Urban–Rural Classification Scheme [14], where urban areas included large metropolitan counties (population ≥ 1 million) and small or medium metropolitan counties (population 50,000-999,999), whereas rural areas referred to nonmetropolitan counties with populations < 50,000. As the U.S. National Center for Health Statistics has not yet released the latest urban and rural data beyond 2020, subsequent analyses will be limited to urbanization data from 1999 to 2020.

### Statistical analysis

Age-adjusted mortality rates (AAMRs) and crude mortality rates per 100,000 population were obtained from the CDC WONDER database for the period 1999–2023. The AAMR was standardized to the 2000 U.S. standard population, in accordance with CDC and NCHS guidelines. To evaluate long-term temporal trends in mortality involving both dementia and DM, we calculated the percentage change in AAMR across the study period. AAMR was used as the primary outcome for all analyses, whereas crude mortality rates were employed in age-stratified analyses to avoid over-adjustment. Mortality rates were summarized annually and further stratified by sex, race or ethnicity, census region, and urbanization level, with 95% confidence intervals (CIs) for each subgroup. Temporal trends in mortality were assessed using the Joinpoint Regression Program [15]. This software fits segmented log-linear regression models to identify statistically significant changes in temporal trends. In the main analysis, the maximum number of joinpoints was set to 4, while a maximum of 3 was used for the sensitivity analysis (1999–2019). The optimal number of joinpoints was determined via permutation tests, requiring a minimum of two observations between consecutive joinpoints. Models were fitted assuming uncorrelated residuals. For each identified segment, the Annual Percent Change (APC) and its 95% CI were calculated. To summarize the overall trend across the entire study period, the AAPC was calculated, with its 95% CI estimated using a parametric method based on the log-linear model. Statistical significance for both APC and AAPC was assessed based on whether the 95% CI excluded zero. Differences in mortality trends between demographic subgroups (e.g., by sex, race/ethnicity, age group, and urbanization) were compared using the test of parallelism in Joinpoint regression. A two-tailed *P* value < 0.05 was considered statistically significant.

Additionally, to project future mortality patterns, an autoregressive integrated moving average (ARIMA) model was employed using time-series data from 1999 to 2019. This model was used to forecast the expected AAMR in subsequent years under the assumption of consistent trend patterns, allowing for a predictive assessment of future disease burden [16]. ARIMA analyses were conducted to evaluate trends in AAMR and death counts by sex. For each series (male, female, and both sexes), ARIMA(p, d,q) models were fitted. The orders (p, d,q) were determined by visual inspection of the autocorrelation function (ACF) and partial autocorrelation function (PACF), and by selecting models that minimized the Akaike Information Criterion (AIC) and Bayesian Information Criterion (BIC). To achieve stationarity, differencing was applied according to the selected d values (1 or 2). Unit root tests, including the Augmented Dickey-Fuller (ADF) test and KPSS test, were performed on the original and differenced series to confirm stationarity. Model residuals were further assessed through ACF and PACF and the Ljung-Box test to evaluate whether any significant autocorrelation remained. To validate the ARIMA model’s predictive accuracy, we trained the model using 1999–2017 data and compared forecasts to the observed 2018–2019 data. The small prediction errors indicate the model captures underlying mortality dynamics and can reliably forecast the near-term trends. Sensitivity analyses were conducted to assess the potential influence of the COVID-19 pandemic on long-term trend estimates. Joinpoint regression was restricted to the pre-pandemic period (1999–2019), and AAPCs with corresponding 95% confidence intervals were recalculated. In addition, expected AAMRs for 2020–2023 were projected based on pre-pandemic trends using a log-linear regression model. Excess mortality was calculated as the difference between actual and expected mortality rates, expressed as a percentage change.

All statistical analyses and visualization were conducted using R software (version 4.3.1) and the Joinpoint Regression Program (version 5.2.0).

## Result

From 1999 to 2023, a total of 749,517 deaths related to DM and dementia were reported in the United States. In 2023 alone, 44,882 deaths were recorded, representing a 294.3% increase compared with 11,382 deaths in 1999 (Table 1). During the same period, the overall AAMR rose from 12.05 (95% CI 11.83–12.28) to 30.95 (95% CI 30.66–31.24) per 100,000 population, with an AAPC of 3.48 (95% CI 1.66–5.32, *P* < 0.05).

**Table 1:** Trends in deaths and age-adjusted mortality rates (AAMR) for DM and dementia in the United States, 1999–2023

Characteristic	Deaths					AAMR		
	Overall	Deaths_1999	Deaths_2023		Percent change	AAMR_1999	AAMR_2023	AAPC (95% CI)
**Both**	749,517	11,382	44,882	294.32		12.05 (11.83 to 12.28)	30.95 (30.66 to 31.24)	3.48 (1.66 to 5.32*)
**Sex**								
Female	446,623	7247	25,002	245.00		11.79 (11.52 to 12.07)	29.24 (28.88 to 29.61)	3.51 (2.50 to 4.52*)
Male	302,894	4135	19,880	380.77		12.26 (11.88 to 12.64)	33.15 (32.68 to 33.62)	3.68 (2.02 to 5.38*)
**Census Region**								
Northeast	119,913	2133	6193	190.34		10.41 (9.97 to 10.85)	22.48 (21.92 to 23.04)	2.94 (1.41 to 4.49*)
Midwest	183,693	2992	9322	211.56		12.91 (12.45 to 13.38)	30.47 (29.85 to 31.09)	3.64 (0.88 to 6.49*)
South	284,885	4368	19,506	346.57		13.39 (12.99 to 13.79)	35.77 (35.27 to 36.28)	3.72 (1.80 to 5.67*)
West	161,026	1889	9861	422.02		10.33 (9.86 to 10.79)	30.43 (29.83 to 31.03)	4.17 (3.01 to 5.34*)
**Race**								
Hispanic	58,532	433	4388	913.39		11.76 (10.64 to 12.89)	34.52 (33.49 to 35.55)	3.01 (0.73 to 5.33*)
NH Black	93,778	1392	5692	308.91		19.29 (18.28 to 20.31)	45.15 (43.96 to 46.34)	2.86 (0.96 to 4.79*)
NH White	570,045	9359	32,503	247.29		11.47 (11.24 to 11.71)	29.55 (29.22 to 29.87)	3.67 (2.69 to 4.65*)
NH Other	25,661	169	2211	1208.28		8.57 (7.24 to 9.89)	22.76 (21.81 to 23.71)	2.65 (0.44 to 4.92*)
**Urbanization** ^**#**^								
Metropolitan	481,670	8856	35,627	302.29		11.65 (11.41 to 11.90)	34.32 (33.99 to 34.66)	4.68 (3.67 to 5.70*)
Nonmetropolitan	129,067	2526	9255	266.39		13.70 (13.17 to 14.24)	43.75 (42.91 to 44.60)	5.22 (4.06 to 6.40*)
**Age Group** ^**##**^								
45–54 years	1313	25	51	104.00		0.07 (0.07 to 0.07)	0.13 (0.13 to 0.13)	1.15 (0.10 to 2.21*)
55–64 years	11,983	143	776	442.66		0.60 (0.60 to 0.60)	1.85 (1.85 to 1.85)	2.94 (1.56 to 4.34*)
65–74 years	75,622	1187	5309	347.26		6.44 (6.44 to 6.44)	15.31 (15.31 to 15.31)	3.39 (2.23 to 4.56*)
75–84 years	273,879	4766	16,948	255.60		38.99 (38.99 to 38.99)	92.27 (92.27 to 92.27)	3.45 (2.48 to 4.44*)
85 + years	386,720	5261	21,798	314.33		126.65 (126.65 to 126.65)	351.87 (351.87 to 351.87)	3.96 (2.88 to 5.04*)

*P < 0.05; ^#^Urbanization data were available only for 1999–2020. For this subgroup, values reported in the columns “Deaths_2023” and “AAMR_2023” correspond to estimates from 2020. Percent change and AAPC for the urbanization subgroup were calculated for the period 1999–2020 only; ^##^AAMRs for age groups were derived from crude rates; NH = non-Hispanic; AAMR = age-adjusted mortality rate per 100,000 population; AAPC=average annual percent change

### Overall age-adjusted trends for DM and dementia-related mortality from 1999 to 2023

**Fig. 1:**
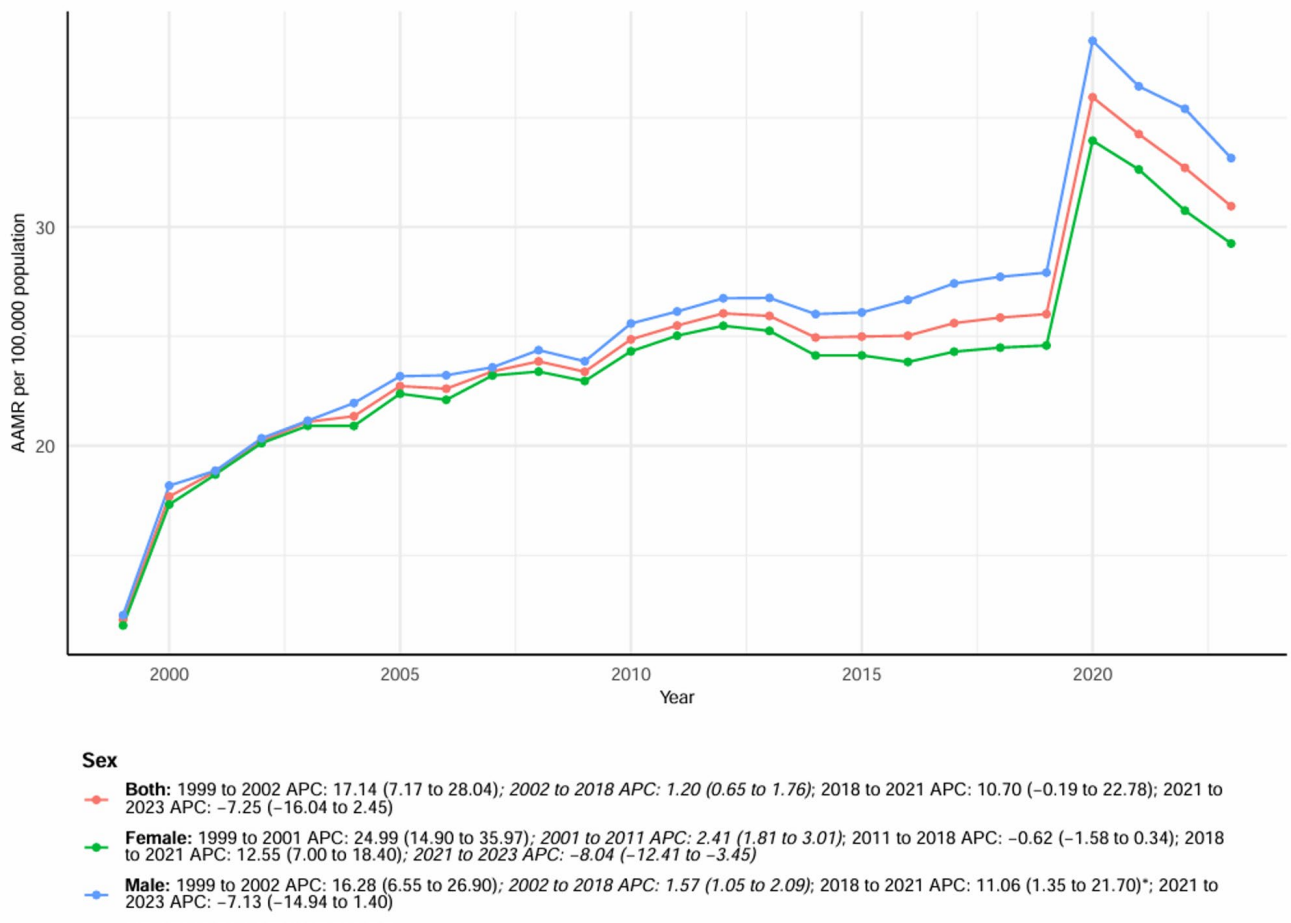
Trends in overall and sex-stratified age-adjusted DM and Dementia-related mortality in the United States from 1999 to 2023. APC = Annual Percentage Change, CI = Confidence Interval. *Indicates that the Annual Percentage Change (APC) is significantly different from zero at α = 0.05

The AAMR rose sharply from 1999 to 2005, with an APC of approximately 9.8%, followed by a period of relative stabilization between 2006 and 2015, during which the increase slowed substantially (APC = 0.5%, *P* > 0.05). From 2015 onward, mortality rates began to climb again, reaching a secondary peak during 2018–2021, before showing a mild decline after 2021 (Fig. 1, Table S1). Overall, despite these fluctuations, the long-term trajectory remained significantly upward, suggesting a sustained increase in DM and dementia-related mortality among individuals over the past two decades.

To assess the potential influence of the COVID-19 pandemic on long-term trends, a sensitivity analysis restricted to the pre-pandemic period (1999–2019) was performed (Table S8). During this period, the AAMR increased from 12.05 to 26.02 per 100,000 population, with an AAPC of 3.48% (95% CI 2.57–4.39, *P* < 0.05), which was nearly identical to the estimate based on the full period (1999–2023). Similar increasing patterns were observed across sex, region, race, and age subgroups. Based on pre-pandemic trends, a log-linear model was used to project the AAMR for 2020–2023 (Table S9). Actual mortality rates exceeded projections from 2020 to 2022 but returned to projected levels by 2023.

To further characterize the contribution of each condition to overall mortality, we examined the proportion of deaths in which DM or dementia was listed as the underlying versus contributing cause. Table S10 presents the annual numbers and percentages of deaths from 1999 to 2023, distinguishing two mutually exclusive categories: (1) DM as the underlying cause with dementia as a contributing cause, and (2) dementia as the underlying cause with DM as a contributing cause. Overall, deaths with dementia as the underlying cause accounted for a larger proportion of total deaths throughout the study period, increasing from 1.04% in 1999 to 6.01% in 2023, whereas deaths with DM as the underlying cause ranged from 2.35% to 4.36%.

### DM and dementia-related mortality stratified by demographics

#### Sex

From 1999 to 2023, both males and females experienced significant increases in DM and dementia-related mortality. However, the test of parallelism indicated that the temporal trends were not parallel between males and females (*P* for parallelism 0.022) (Table S7). The number of deaths among males rose from 4,135 in 1999 to 19,880 in 2023, whereas female deaths increased from 7,247 to 25,002 during the same period. The AAMR for males increased from 12.26 (95% CI: 11.88–12.64) to 33.15 (95% CI: 32.68–33.62) per 100,000 population, while that for females rose from 11.79 (95% CI: 11.52–12.07) to 29.24 (95% CI: 28.88–29.61). Both trends were statistically significant, with an AAPC of 3.68% (95% CI: 2.02–5.38) for males and 3.51% (95% CI: 2.50–4.52) for females (Table 1; Fig. 1, Figure S1). The mortality rates for males remained consistently higher than those for females across all age groups. The bilateral pattern demonstrates a clear left–right symmetry, with males showing greater death counts and AAMRs in each corresponding age category (Figure S1). The male AAMR rose sharply from 1999 to 2010, then maintained a slower yet steady increase until 2019, followed by minor fluctuations thereafter. Female AAMRs showed a smoother but sustained upward trend throughout the period. Despite interannual variability, both sexes demonstrated a statistically significant overall upward trajectory (*P* < 0.05) (Fig. 1).

#### Race

Between 1999 and 2023, the AAMR for DM and dementia varied substantially across racial and ethnic groups in the United States (Table 1, Table S2, Figure S2). Overall, NH Black individuals exhibited the highest mortality burden throughout the study period, followed by Hispanic, NH White, and NH Other races. In 1999, the AAMR was 19.3 per 100,000 among NH Blacks compared to 11.5 in NH Whites, 11.8 in Hispanics, and 8.6 in NH Others. By 2023, the rates rose to 45.2 (95% CI: 44.0-46.3), 34.5 (33.5–35.6), 29.5 (29.2–29.9), and 22.8 (21.8–23.7) per 100,000, respectively.

Joinpoint analysis demonstrated that all racial groups experienced a significant upward trend in mortality over the study period. Parallelism tests revealed non-parallel trends between Hispanic and non-Hispanic Black individuals (*P* for parallelism < 0.001), as well as between non-Hispanic Black and non-Hispanic White individuals (*P* for parallelism 0.020), whereas trends among other racial/ethnic pairwise comparisons were statistically parallel (*P* for parallelism > 0.05) (Table S7). The AAPC was highest among NH Whites (3.67%, 95% CI: 2.69% to 4.65%), followed by Hispanics (3.01%, 0.73% to 5.33%), NH Blacks (2.86%, 0.96% to 4.79%), and NH Others (2.65%, 0.44% to 4.92%). Overall, the AAMR increased most rapidly from 1999 to 2005, slowed between 2005 and 2013, and then maintained a moderate upward trajectory thereafter, with NH Black individuals consistently showing the highest mortality rate across all periods.

#### Age

**Fig. 2:**
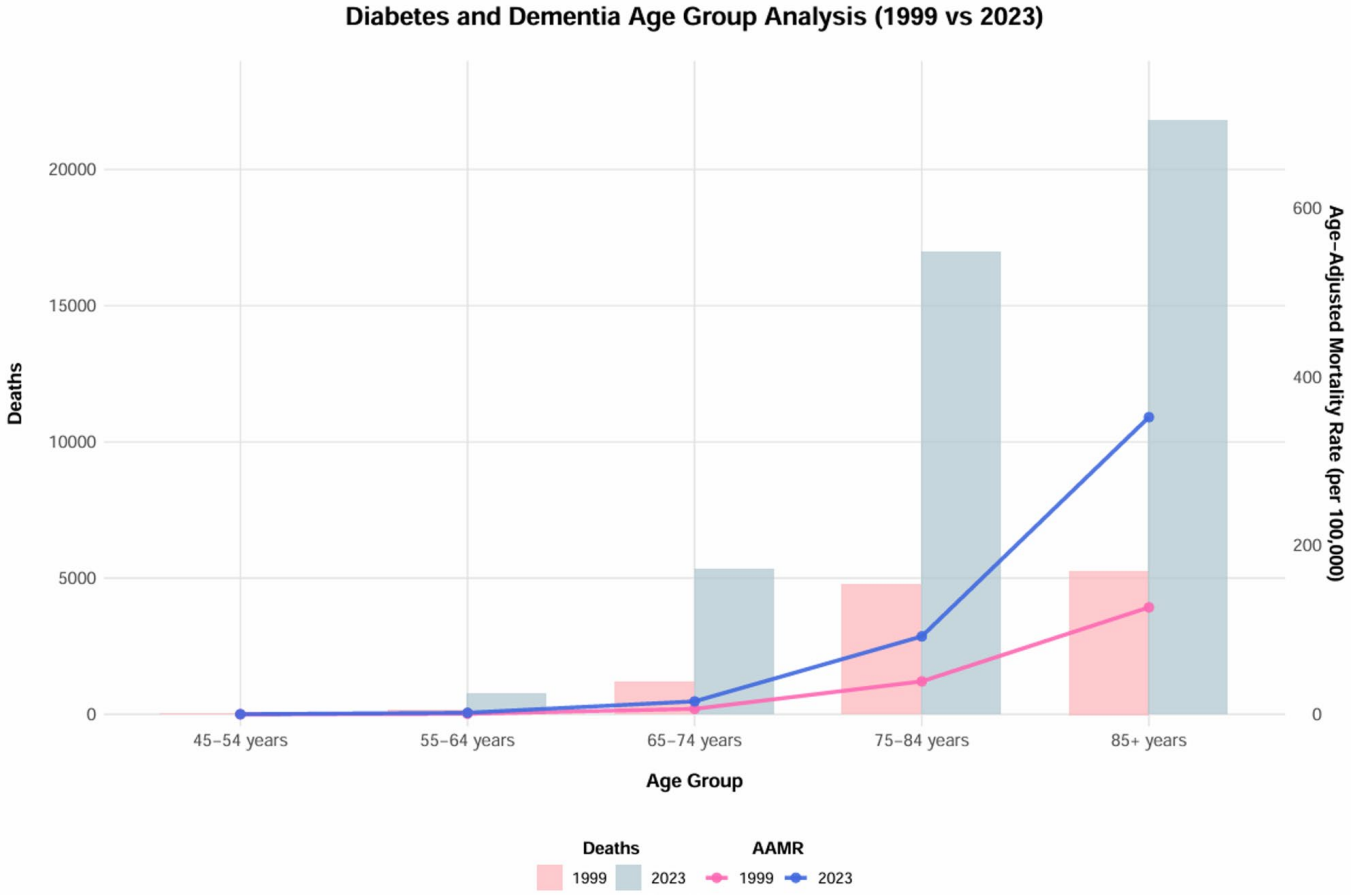
Comparison of deaths and age-adjusted mortality rates for DM and dementia by age group, United States, 1999 and 2023. Note: Bars represent the number of deaths (left axis) and lines represent the age-adjusted mortality rates (AAMRs) per 100,000 population (right axis). AAMR = age-adjusted mortality rate

From 1999 to 2023, age-specific crude mortality rates for diabetes and dementia-related conditions increased exponentially with age, and all age groups exhibited broadly similar temporal patterns over the study period (Table S7). The 85 + year group reaching a mean of 272.5 per 100,000 (95% CI: 248.9-296.1), approximately 2725 times higher than the 0.1 per 100,000 (95% CI: 0.1–0.1) observed in the 45–54 year group (Table S3). All age groups demonstrated significant upward trends, with AAPC rising progressively from 1.15% (95% CI: 0.10%-2.21%) in the 45–54 year group to 3.96% (95% CI: 2.88%–5.04%) in the 85 + year group (Table 1, Figure S3). Rates rose steadily before 2019, followed by a marked acceleration between 2019 and 2021 and a subsequent slowdown from 2022 to 2023 across all age groups. This pattern was most prominent in the 85 + year group, where the rate surged from 290.7 per 100,000 in 2019 to 396.8 per 100,000 in 2020 before declining to 351.9 per 100,000 in 2023; similar, though less pronounced, shifts occurred in younger age groups (Table S3, Fig. 2).

#### State and census region

**Fig. 3:**
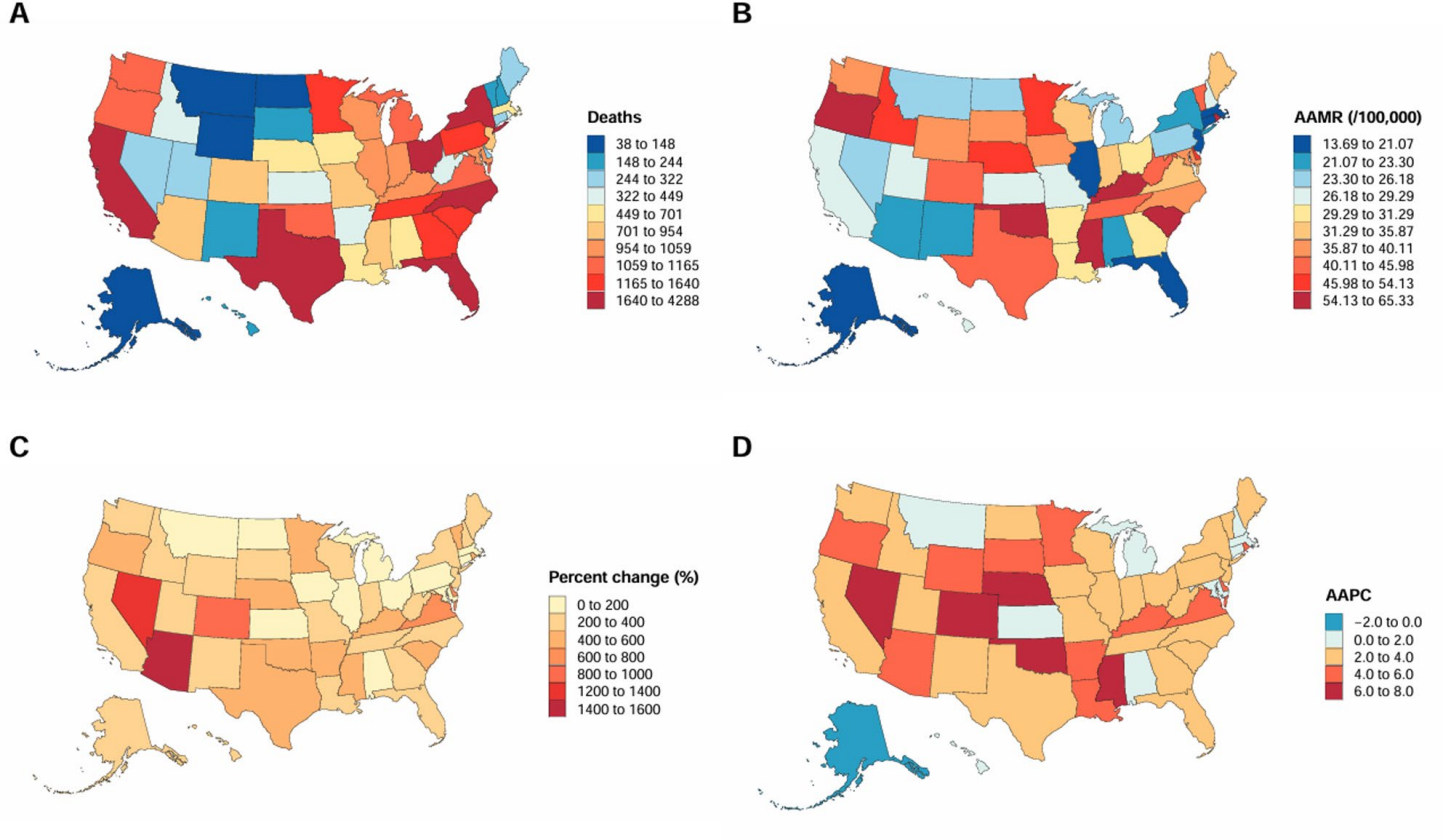
DM and dementia-associated age-adjusted death rates per 100,000, among middle-aged and elderly adults categorized by states in the United States, 1999 to 2023. Note: *Map lines delineate study areas and do not necessarily depict accepted national boundaries

Substantial geographic variation in diabetes and dementia-related mortality was observed across states and census regions from 1999 to 2023. State-level mean AAMRs ranged from 12.4 per 100,000 (95% CI: 10.1–14.7) in Nevada to 38.8 per 100,000 (95% CI: 35.2–42.3) in Vermont, a more than threefold difference (Table S4, Fig. 3B). The highest absolute number of deaths occurred in California, Florida, Texas, and New York, whereas the highest AAMRs were observed in Oklahoma, Minnesota, South Carolina, Kentucky, and Rhode Island (Fig. 1A, B). Percent changes in death counts were largest in most midwestern states, with Texas and Florida recording the greatest increases (Fig. 1C). States with the highest average annual percent changes (AAPCs), reaching 6.0%-8.0%, were located primarily in the Midwest and South (Fig. 1D).

By census region, most pairwise regional comparisons exhibited non-parallel temporal trends, whereas only the trends between the South and West were statistically parallel (Table S7). Overall, the Midwest recorded the highest overall AAMR at 26.9 per 100,000 (95% CI: 26.1–27.7), followed by the South at 26.8 per 100,000 (95% CI: 26.2–27.4), the West at 25.4 per 100,000 (95% CI: 24.7–26.1), and the Northeast at 20.6 per 100,000 (95% CI: 19.8–21.4) (Table S6). A significant increase was observed from 1999 to 2010, followed by a generally slower growth rate from 2010 to 2019. After 2020, negative growth emerged across regions, with the most pronounced declines occurring in the West and Northeast (Table S6, Figure S6). Urban-rural analyses were restricted to the period from 1999 to 2020 due to the availability of urbanization classification data. During this period, pronounced urban-rural disparities in mortality trends were observed, with non-parallel temporal trends identified between metropolitan and non-metropolitan areas (Table S7). with nonmetropolitan areas demonstrating a mean AAMR of 28.6 per 100,000 (95% CI: 28.0-29.2) compared with 23.8 per 100,000 (95% CI: 23.4–24.2) in metropolitan areas, accompanied by a steeper AAPC of 5.22% (95% CI: 4.06%-6.40%) versus 4.68% (95% CI: 3.67%-5.70%) (Table 1, Table S5, Figure s5).

### Prediction of mortality in United States during 1999–2029

The selected ARIMA models for each series were as follows: male AAMR ARIMA(1,2,0), female AAMR ARIMA(1,2,0), both-sexes AAMR ARIMA(1,2,0); male deaths ARIMA(0,1,0), female deaths ARIMA(1,2,0), and both-sexes deaths ARIMA(0,1,0). Differencing was applied according to the respective d values to achieve stationarity. The original series were tested for stationarity using Augmented Dickey-Fuller (ADF) and KPSS tests, which indicated non-stationarity. Consequently, differencing was applied according to the selected ARIMA model orders. Unit root tests (ADF and KPSS) were then performed on the differenced series, confirming that all series satisfied the stationarity assumption before model fitting. Residual diagnostics were conducted to assess model adequacy. Examination of residual autocorrelation and partial autocorrelation functions (ACF/PACF) revealed no significant patterns, and the Box-Ljung test indicated that residuals were approximately white noise (*P* > 0.05), suggesting an adequate model fit.

**Fig. 4:**
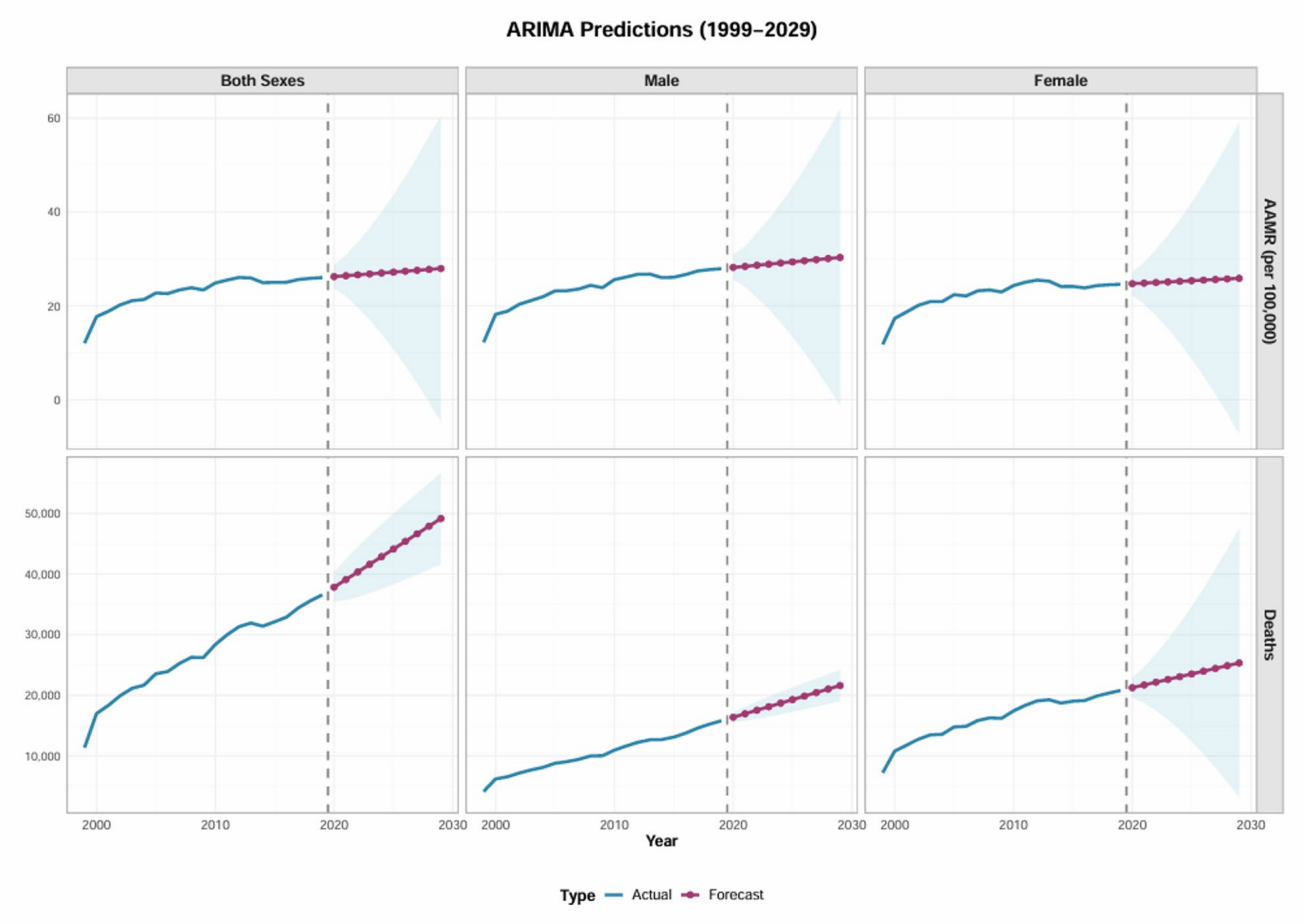
ARIMA-based forecast of age-adjusted mortality rates for DM and dementia in the United States, 1999–2029. Note: The solid line shows observed AAMRs (1999-2019) and the dashed line indicates ARIMA-predicted values through 2029 with 95% confidence intervals. AAMR = age-adjusted mortality rate

Time series analysis using ARIMA models on 1999–2019 data forecasted sustained elevation in diabetes and dementia-related mortality to 2029 (Fig. 4). The overall AAMR is expected to increase from 26.02 per 100,000 in 2019 to 27.96 per 100,000 in 2029, with 95% confidence intervals ranging from − 4.68 to 60.59 per 100,000. Annual deaths are projected to rise from 41,611 in 2023 to 49,168 in 2029. Male-specific projections indicate an increase in AAMR from 27.91 per 100,000 in 2019 to 30.31 per 100,000 in 2029, with annual deaths rising from 15,787 to 21,613. For females, the AAMR is projected to increase from 24.58 per 100,000 in 2019 to 25.86 per 100,000 in 2029, with deaths increasing from 20,786 to 25,339 (Fig. 4).

To further assess the robustness of the ARIMA models and their ability to capture pre-pandemic temporal dynamics, a robustness analysis was conducted using data from 1999 to 2017, with out-of-sample validation against observed values in 2018–2019 (Table S11). The predicted values for 2018–2019 were highly consistent with the observed data, with all observations falling within the 95% prediction intervals. External validation errors, as assessed by RMSE, MAE, and MAPE (%), were small and within acceptable ranges across sex-specific and overall series, indicating good predictive performance. These findings suggest that the ARIMA models are robust and capable of capturing the underlying temporal dynamics of the outcomes prior to major external disruptions.

## Discussion

Using nationwide mortality data from the CDC WONDER Multiple Cause of Death database, this study characterized long-term trends in deaths in which both diabetes mellitus (DM) and dementia were recorded on the death certificate among U.S. adults aged ≥ 45 years from 1999 to 2023. We observed several important findings. First, the AAMR increased 2.57-fold over the study period, with a sustained upward trajectory from 1999 to 2021 followed by a decline toward 2023. Sensitivity analyses restricted to the pre-pandemic period (1999–2019) yielded nearly identical trend estimates, indicating that the long-term increase preceded the COVID-19 pandemic. Second, males consistently exhibited higher AAMRs than females throughout the study. Third, across racial and ethnic groups, NH Black individuals had the highest DM and dementia-related mortality. Fourth, mortality rates in rural areas remained persistently higher than those in urban regions. Fifth, AAMR exhibited marked geographic variation. States in the upper 90th percentile had an average AAMR of 36.2 per 100,000, approximately 2.5 times higher than the 14.6 per 100,000 observed in states in the lower 10th percentile. Moreover, among deaths involving both conditions, dementia was more frequently recorded as the underlying cause than diabetes (68.9% vs. 31.1% of the total 422,359 deaths). Over the study period, the annual number of deaths with dementia as the underlying cause increased approximately 5.8-fold, compared with a 1.8-fold increase for diabetes.

The long-term upward trend in mortality observed in this study is consistent with the findings reported by Waqas et al.^10^. However, sensitivity analyses restricted to 1999–2019 demonstrated a comparable average annual percent change, indicating that the sustained increase preceded the COVID-19 pandemic and reflects an underlying trajectory of metabolic–cognitive multimorbidity. We observed a pronounced rise in mortality between 2018 and 2021, with a sharp peak centered in 2020. Excess mortality analyses showed that observed AAMRs exceeded projected values during 2020–2022 but returned toward expected levels by 2023, suggesting a transient period effect rather than a permanent structural shift. Several mechanisms may explain this temporary surge. Patients with diabetes faced a 30%-50% increased risk of severe outcomes due to virus-induced hyperglycemia, cytokine storm, and coagulopathy, resulting in 18.4–47.6% excess mortality [17, 18]. Individuals with dementia experienced 8.8–14.2% excess deaths from disrupted long-term care, undetected infections, and pneumonia complications [19]. Healthcare overcrowding, reduced medication access, lockdown-associated depression, and disordered eating during the pandemic further compromised the management of diabetes and dementia. Multiple studies reported significant elevations in glycated hemoglobin, total cholesterol, and triglycerides among patients with diabetes in 2020-2021 [20, 21]. Furthermore, COVID-19 was frequently recorded as the primary cause of death, potentially obscuring the contributions of diabetes and dementia. By including only those death records in which both diabetes and dementia were listed, our study may have reduced potential attribution bias during the COVID-19 pandemic period, thereby providing a more robust assessment of mortality patterns associated with these conditions during 2018–2021. The substantial decline from 2021 to 2023 likely reflects widespread vaccination, falling hospitalization rates, and the restoration of healthcare capacity [22]. To explore near-term patterns beyond the pandemic period, ARIMA models trained exclusively on pre-pandemic data were used to generate projections through 2029, and model performance in the holdout validation further supported the stability of the forecasting approach. These forecasts suggest a continued gradual increase in mortality; however, they represent statistical extrapolations of historical patterns rather than mechanistic predictions. Because the models do not explicitly incorporate potential changes in diabetes prevalence, population aging, diagnostic practices, or healthcare policy, the estimates should be interpreted as indicative of expected trajectories under the continuation of historical patterns rather than precise predictions of future burden. Taken together, the findings indicate that COVID-19 acted as an acute period shock superimposed on a pre-existing upward trajectory, while the broader long-term burden of co-occurring diabetes and dementia remains substantial.

Our finding of higher DM and dementia-related mortality in males is consistent with decades of evidence from the United States showing greater male vulnerability across chronic diseases [23]. It may be attributed to lower healthcare-seeking behaviour among men, resulting in delayed diabetes management and dementia screening, as well as higher prevalence of adverse lifestyle factors such as smoking and excessive alcohol consumption [24]. These behavioural differences likely exacerbate vascular and neurodegenerative pathways, amplifying mortality risk in the presence of diabetes.

Our findings revealed notable racial and ethnic disparities in diabetes and dementia-related mortality. Throughout the study period, NH Black individuals consistently showed the highest age-adjusted mortality rates, followed by Hispanic and NH White populations. These results align with prior evidence suggesting that both diabetes and cognitive disorders are more prevalent among NH Black adults [25, 26]. Several factors may explain this disparity. Lower socioeconomic status, reduced health literacy, and limited insurance coverage can restrict access to early diagnosis and appropriate management [27]. Previous research also indicates that NH Black adults have lower rates of diabetes control and self-care adherence compared with White adults [28]. Furthermore, environmental exposures and geographical residence may contribute to the observed differences. Living in areas with higher pollution levels, smoking prevalence, or reduced healthcare resources may compound existing health inequities. these findings emphasize that racial disparities in diabetes-dementia comorbidity mortality likely stem from a combination of biological, behavioral, and structural determinants, reinforcing the need for tailored public health strategies targeting high-risk populations.

In addition to sociodemographic disparities, marked geographic variations were observed, reflecting uneven mortality patterns across different U.S. regions and levels of urbanization. The AAMRs were persistently higher in the South and Midwest compared with the Northeast, with the gap between states reaching approximately 3. Moreover, individuals living in rural areas consistently exhibited greater mortality than those in urban settings. These findings mirror previous national epidemiological reports showing that the highest burdens of diabetes and cognitive impairment cluster in socioeconomically disadvantaged regions [29, 30]. Multiple reasons likely contribute to these disparities. Rural and southern regions often face shortages of healthcare professionals, lower density of endocrinology and neurology services, and limited access to early screening or chronic disease management [31]. Socioeconomic disadvantages, including poverty, lower educational attainment, and lack of health insurance, further restrict timely care [32, 33]. Environmental and lifestyle factors, such as higher prevalence of obesity, physical inactivity, tobacco use, and poor dietary habits, may also exacerbate regional inequalities [34]. Regionally tailored public health initiatives—such as expanding telemedicine networks, strengthening community-based chronic disease programs, and improving healthcare infrastructure in underserved areas—are essential to mitigate disparities and improve long-term outcomes among middle-aged and older adults affected by both diabetes and dementia.

Our study offers important insights into the evolving burden of DM and dementia-related mortality among middle-aged and older adults in the United States. The observed increase in AAMR highlights the urgent need for coordinated preventive strategies that address both metabolic and cognitive health within an aging population. Early identification and integrated management of diabetes may reduce the long-term risk of cognitive decline and dementia, underscoring the value of multidisciplinary collaboration between primary care, endocrinology, and neurology. Public health policies should prioritize health education, behavioral interventions, and equitable access to care, particularly in high-risk regions and underserved rural areas. From a policy perspective, these findings emphasize the importance of strengthening surveillance systems, promoting telemedicine, and improving continuity of care for patients with chronic multimorbidity. Integrating diabetes management with cognitive screening in community health programs could be an effective strategy to curb rising mortality.

## Limitations

Our study has several limitations. First, the reliance on death certificates and ICD-10 coding introduces the possibility of diagnostic misclassification, miscoding, omission, and data transcription errors. Notably, the sharp jump in mortality centered in 2020 must be interpreted with caution. This increase may reflect “coding inflation” during the COVID-19 era. Furthermore, our excess mortality analysis—showing an overall excess AAMR of 22.7% in 2020—suggests that this spike likely reflects a concentration of deaths among highly vulnerable individuals. Patients with both diabetes and dementia were at an exceptionally high risk for severe COVID-19 outcomes and healthcare disruptions, potentially leading to “mortality displacement,” where deaths that might have occurred in later years were shifted forward to the early pandemic period. Although the ICD-10 system provides a standardized framework for mortality surveillance, these potential inaccuracies and temporal shifts in coding practices cannot be fully eliminated. Second, the database lacked key laboratory indicators and detailed information on comorbidities, lifestyle factors, and treatment history, which limited our ability to comprehensively assess disease severity and treatment response. Third, given the large number of subgroup and pairwise comparisons, including census regions, race, age, sex, and urbanization levels, formal adjustments for multiple testing were not applied, which may increase the risk of type I errors. However, the analyses were primarily descriptive rather than confirmatory. Future studies using independent datasets are warranted to confirm these trends. Finally, data on urbanization were not updated beyond 2020 in the CDC WONDER database. As a result, urban-rural analyses were limited to the period from 1999 to 2020, and post-2020 urban-rural patterns could not be evaluated in this study. Future studies incorporating updated urbanization classification data will be needed to assess whether urban-rural disparities have evolved in more recent years.

## Conclusions

This nationwide study utilized the CDC WONDER multiple cause-of-death database to examine long-term trends in DM and dementia-related mortality among middle-aged and older adults from 1999 to 2023. We identified a sustained increase in age-adjusted mortality, with significant disparities across sex, race, and geographic regions. These findings underscore the complex interaction between metabolic and cognitive health and highlight persistent health inequities within the United States. Future research should explore approaches to improve healthcare accessibility among high-risk populations and further investigate the behavioral, socioeconomic, and systemic determinants underlying these trends, striving toward more equitable healthcare delivery and enhanced support for affected individuals.

## Supplementary Information

Supplementary Material 1.

## Data Availability

All data used in this study are publicly available through the Centers for Disease Control and Prevention (CDC) Wide-ranging Online Data for Epidemiologic Research (WONDER) platform. Mortality data were obtained from the Multiple Cause of Death database ( [https://wonder.cdc.gov](https:/wonder.cdc.gov/mcd.html) ).
