# Supplementary material for: Long-term trends in DM and dementia-related mortality among middle-aged and elderly adults in the United States, 1999–2023: a nationwide population-based study
Source: BMC Public Health. 2026 Apr 14;26:1668. doi: 10.1186/s12889-026-27257-9 (PMC13195832; doi:10.1186/s12889-026-27257-9)
Supplement: Supplementary file 1 — Supplementary Material 1. [file 12889_2026_27257_MOESM1_ESM.docx]

**Supplementary Table 1.** Overall and sex-stratified DM and Dementia-related AAMR per 100,000 in the United States from 1999 to 2023.

| **Age-Adjusted Rate /100,000 (95% CI)** | | | |
| --- | --- | --- | --- |
| **Year** | **Overall** | **Male** | **Female** |
| 1999 | 12.1 (11.8-12.3) | 12.3 (11.9-12.6) | 11.8 (11.5-12.1) |
| 2000 | 17.7 (17.4-18.0) | 18.2 (17.7-18.6) | 17.3 (17.0-17.7) |
| 2001 | 18.8 (18.6-19.1) | 18.9 (18.4-19.3) | 18.7 (18.4-19.0) |
| 2002 | 20.2 (20.0-20.5) | 20.3 (19.9-20.8) | 20.1 (19.8-20.5) |
| 2003 | 21.1 (20.8-21.4) | 21.1 (20.7-21.6) | 20.9 (20.6-21.3) |
| 2004 | 21.3 (21.1 - 21.6) | 21.9 (21.5-22.4) | 20.9 (20.5-21.3) |
| 2005 | 22.7 (22.4-23.0) | 23.2 (22.7-23.7) | 22.4 (22.0-22.7) |
| 2006 | 22.6 (22.3-22.9) | 23.2 (22.7-23.7) | 22.1 (21.7-22.5) |
| 2007 | 23.4 (23.1-23.7) | 23.6 (23.1-24.1) | 23.2 (22.8-22.8) |
| 2008 | 23.9 (23.6-24.1) | 24.4 (23.9-24.8) | 23.4 (23.0-23.7) |
| 2009 | 23.4 (23.1-23.7) | 23.9 (23.4-24.3) | 23.0 (22.6-23.3) |
| 2010 | 24.9 (24.6-25.2) | 25.6 (25.1-26.1) | 24.3 (23.9-24.7) |
| 2011 | 25.5 (25.2-25.8) | 26.1 (25.7-26.6) | 25.0 (24.7-25.4) |
| 2012 | 26.0 (25.8-26.3) | 26.7 (26.3-27.2) | 25.5 (25.1 - 25.9) |
| 2013 | 25.9 (25.6-26.2) | 26.8 (26.3-27.2) | 25.2 (24.9 - 25.6) |
| 2014 | 24.9 (24.7-25.2) | 26.0 (25.6-26.5) | 24.1 (23.8 - 24.5) |
| 2015 | 25.0 (24.7-25.3) | 26.1 (25.6-26.5) | 24.1 (23.8-24.5) |
| 2016 | 25.0 (24.8 - 25.3) | 26.7 (26.2-27.1) | 23.8 (23.5-24.2) |
| 2017 | 25.6 (25.3-25.9) | 27.4 (27.0-27.9) | 24.3 (24.0-24.6) |
| 2018 | 25.9 (25.6-26.1) | 27.7 (27.3-28.2) | 24.5 (24.1-24.8) |
| 2019 | 26.0 (25.7-26.3) | 27.9 (27.5-28.4) | 24.6 (24.2-24.9) |
| 2020 | 35.9 (35.6-36.2) | 38.5 (38.0-39.0) | 33.9 (33.6-34.3) |
| 2021 | 34.2 (33.9-34.6) | 36.4 (35.9-36.9) | 32.6 (32.2-33.0) |
| 2022 | 32.7 (32.4-33.0) | 35.4 (34.9-35.9) | 30.7 (30.4 –31.1) |
| 2023 | 31.0 (30.7-31.2) | 33.1 (32.7-33.6) | 29.2 (28.9 –29.6) |
| **Total** | 25.3 (24.5-26.1) | 26.9 (26.0 –27.8) | 23.6 (23.0 –24.2) |

AAMR: age-adjusted mortality rate; CI: confidence interval

| **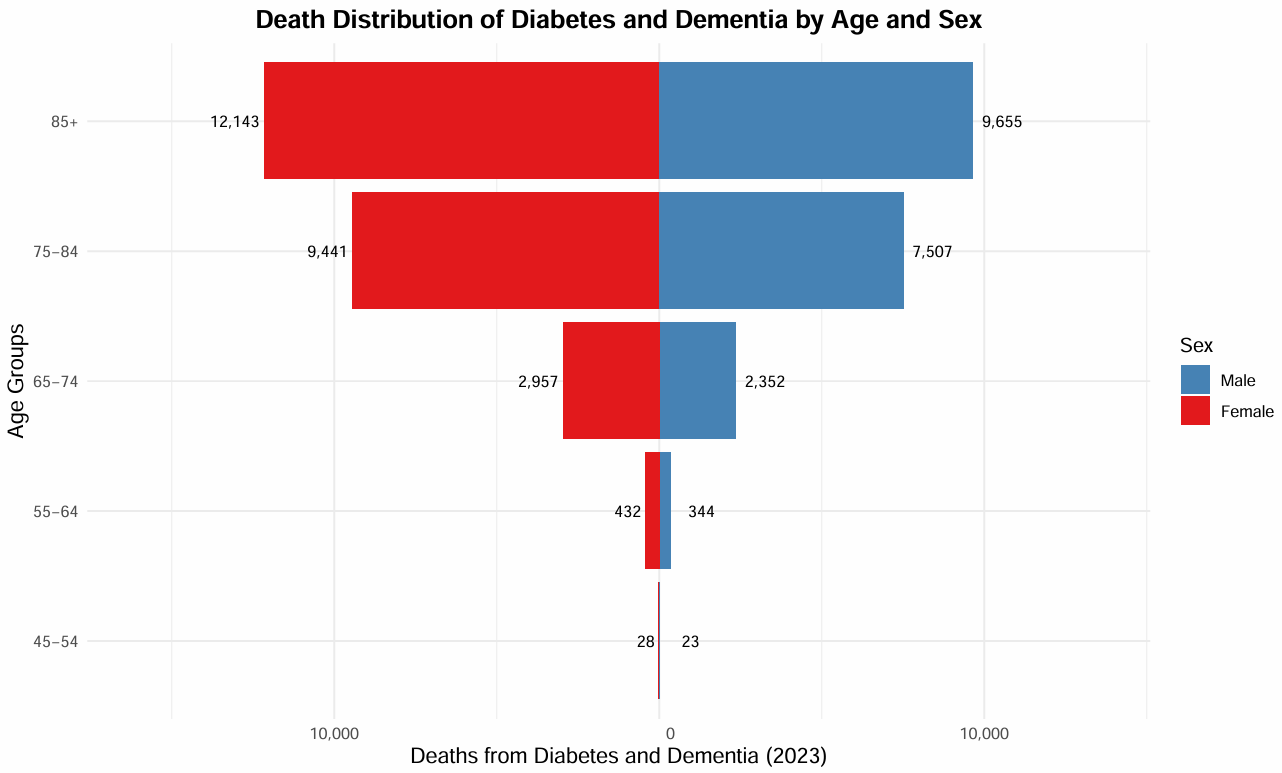**  A | **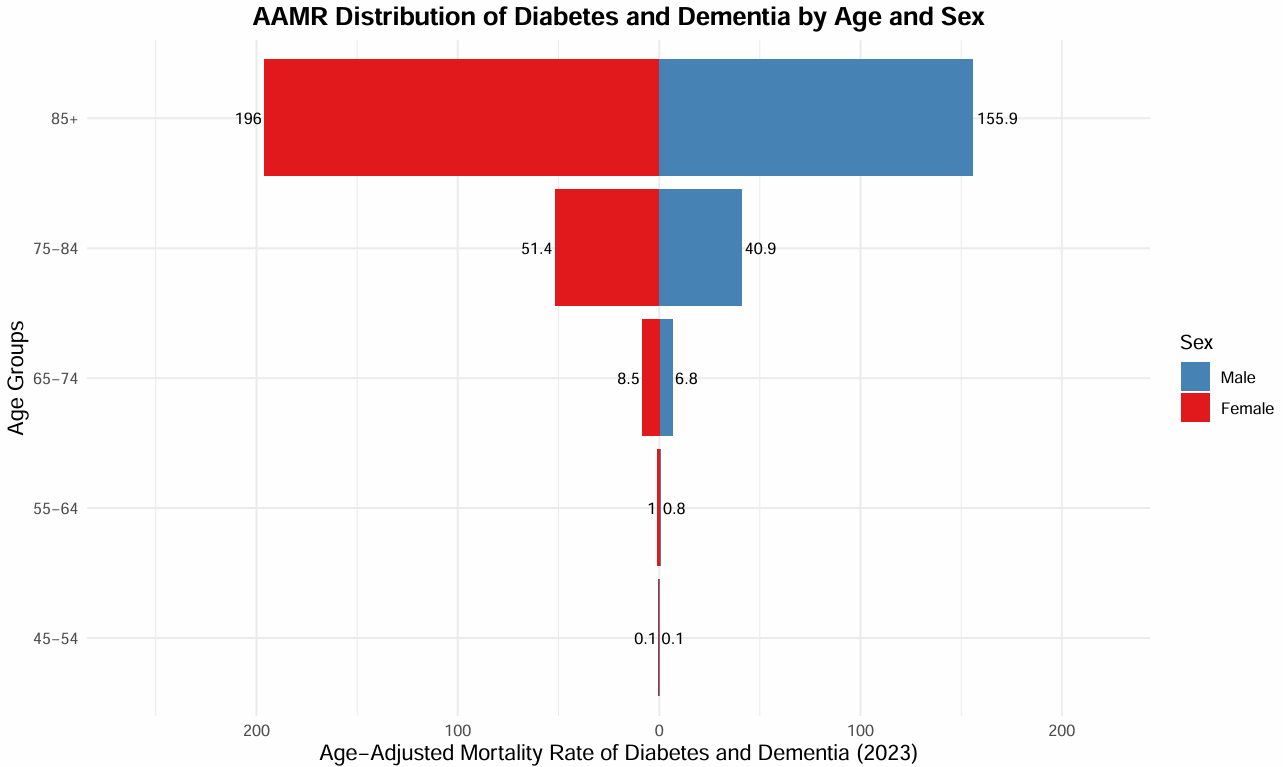**  B |
| --- | --- |

**Supplementary Figure 1.** Sex-specific distribution of deaths (A) and age-adjusted mortality rates (B) for dementia associated with diabetes by age group, United States, 2023.
*Note:* Bars extend left for females and right for males. AAMR = age-adjusted mortality rate.

**Supplementary Table 2.** DM and Dementia-related mortality per 100,000 stratified by race of death in the United States from 1999 to 2023.

| Age-Adjusted Rate /100,000 (95% CI) | | | | |
| --- | --- | --- | --- | --- |
| Year | **Hispanic** | **NH Black** | **NH White** | **NH Other** |
| 1999 | 11.8 (10.6-12.9) | 19.3 (18.3-20.3) | 11.5 (11.2-11.7) | 8.6 (7.2-9.9) |
| 2000 | 17.1 (15.8-18.4) | 29.0 (27.8-30.2) | 16.8 (16.5-17.1) | 13.1 (11.5-14.7) |
| 2001 | 20.0 (18.6-21.4) | 30.8 (29.5-32.1) | 17.7 (17.4-18.0) | 15.6 (14.0-17.2) |
| 2002 | 20.6 (19.2-21.9) | 34.6 (33.3-36.0) | 19.0 (18.7-19.3) | 14.6 (13.1-16.1) |
| 2003 | 22.3 (20.9-23.7) | 36.8 (35.4-38.2) | 19.8 (19.5-20.1) | 15.6 (14.1-17.1) |
| 2004 | 23.0 (21.7-24.4) | 37.1 (35.7-38.5) | 20.0 (19.7-20.3) | 15.1 (13.6-16.6) |
| 2005 | 24.5 (23.1-25.8) | 39.7 (38.3-41.1) | 21.2 (20.9-21.5) | 19.2 (17.6-20.8) |
| 2006 | 24.7 (23.4-26.1) | 38.8 (37.4-40.2) | 21.2 (20.9-21.5) | 17.6 (16.1-19.1) |
| 2007 | 25.3 (24.0-26.7) | 40.1 (38.8-41.5) | 21.9 (21.6-22.2) | 19.5 (18.0-21.0) |
| 2008 | 25.9 (24.6-27.2) | 38.8 (37.5-40.2) | 22.5 (22.2-22.8) | 18.7 (17.2-20.1) |
| 2009 | 26.6 (25.3-27.8) | 37.9 (36.6-39.3) | 21.9 (21.6-22.2) | 19.8 (18.4-21.3) |
| 2010 | 29.0 (27.7-30.4) | 38.7 (37.3-40.0) | 23.4 (23.1-23.7) | 21.6 (20.1-23.0) |
| 2011 | 30.2 (28.9-31.5) | 40.3 (39.0-41.6) | 23.9 (23.6-24.2) | 20.7 (19.3-22.0) |
| 2012 | 30.4 (29.2-31.6) | 41.5 (40.1-42.8) | 24.3 (24.0-24.6) | 21.9 (20.5-23.2) |
| 2013 | 32.6 (31.3-33.8) | 39.3 (38.0-40.5) | 24.2 (23.9-24.5) | 21.7 (20.4-23.0) |
| 2014 | 30.3 (29.1-31.5) | 37.3 (36.1-38.5) | 23.5 (23.2-23.8) | 19.8 (18.6-21.0) |
| 2015 | 29.2 (28.1-30.3) | 37.0 (35.8-38.2) | 23.5 (23.2-23.8) | 21.0 (19.8-22.2) |
| 2016 | 29.2 (28.1-30.3) | 37.2 (36.1-38.4) | 23.6 (23.3-23.9) | 19.9 (18.8-21.1) |
| 2017 | 28.4 (27.4-29.4) | 37.6 (36.4-38.7) | 24.3 (24.0-24.6) | 20.1 (19.0-21.2) |
| 2018 | 29.6 (28.6-30.7) | 36.8 (35.7-37.9) | 24.5 (24.2-24.8) | 21.0 (19.9-22.1) |
| 2019 | 29.9 (28.9-31.0) | 36.8 (35.7-37.9) | 24.8 (24.5-25.1) | 19.8 (18.8-20.9) |
| 2020 | 46.2 (45.0-47.5) | 56.6 (55.3-58.0) | 32.9 (32.6-33.2) | 28.7 (27.5-29.9) |
| 2021 | 39.3 (38.2-40.5) | 50.6 (49.3-51.9) | 32.4 (32.0-32.7) | 26.4 (25.3-27.5) |
| 2022 | 39.2 (38.1-40.4) | 47.6 (46.3-48.8) | 30.9 (30.6-31.2) | 24.6 (23.6-25.6) |
| 2023 | 34.5 (33.5-35.6) | 45.2 (44.0-46.3) | 29.5 (29.2-29.9) | 22.8 (21.8-23.7) |
| Total | 28.5 (27.3-29.7) | 38.6 (37.4-39.8) | 23.3 (22.9-23.7) | 19.7 (18.8-20.6) |

AAMR: age-adjusted mortality rate; CI: confidence interval


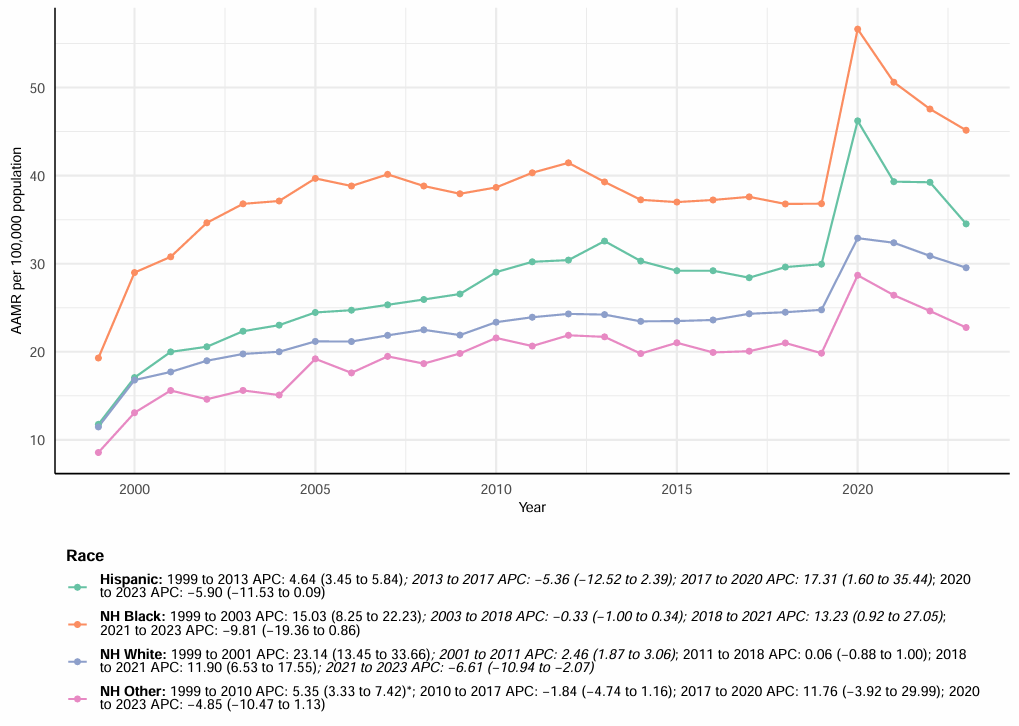


**Supplementary Figure 2.** Trends in Diabetes and Dementia-related mortality stratified by the Race groups in the United States from 1999 to 2023.

APC = Annual Percentage Change, CI = Confidence Interval.

*Indicates that the Annual Percentage Change (APC) is significantly different from zero at α = 0.05.

**Supplementary Table 3.** DM and Dementia-related mortality per 100,000 stratified by age group of death in the United States from 1999 to 2023.

| Crude Rate /100,000 (95% CI) | | | | | |
| --- | --- | --- | --- | --- | --- |
| Year | **45–54 years** | **55–64 years** | **65–74 years** | **75–84 years** | **85+ years** |
| 1999 | 0.1 (0.1-0.1) | 0.6 (0.6-0.6) | 6.4 (6.4-6.4) | 39.0 (39.0-39.0) | 126.6 (126.6-126.6) |
| 2000 | 0.1 (0.1-0.1) | 1.0 (1.0-1.0) | 9.3 (9.3-9.3) | 56.5 (56.5-56.5) | 188.0 (188.0-188.0) |
| 2001 | 0.1 (0.1-0.1) | 1.1 (1.1-1.1) | 9.9 (9.9-9.9) | 59.9 (59.9-59.9) | 200.7 (200.7-200.7) |
| 2002 | 0.1 (0.1-0.1) | 1.0 (1.0-1.0) | 10.8 (10.8-10.8) | 63.8 (63.8-63.8) | 216.9 (216.9-216.9) |
| 2003 | 0.1 (0.1-0.1) | 1.2 (1.2-1.2) | 10.9 (10.9-10.9) | 66.6 (66.6-66.6) | 227.3 (227.3-227.3) |
| 2004 | 0.1 (0.1-0.1) | 1.1 (1.1-1.1) | 10.7 (10.7-10.7) | 68.1 (68.1-68.1) | 230.1 (230.1-230.1) |
| 2005 | 0.1 (0.1-0.1) | 1.1 (1.1-1.1) | 11.2 (11.2-11.2) | 72.2 (72.2-72.2) | 247.1 (247.1-247.1) |
| 2006 | 0.1 (0.1-0.1) | 1.3 (1.3-1.3) | 11.0 (11.0-11.0) | 71.0 (71.0-71.0) | 247.6 (247.6-247.6) |
| 2007 | 0.1 (0.1-0.1) | 1.3 (1.3-1.3) | 11.3 (11.3-11.3) | 74.2 (74.2-74.2) | 254.8 (254.8-254.8) |
| 2008 | 0.1 (0.1-0.1) | 1.2 (1.2-1.2) | 11.3 (11.3-11.3) | 75.0 (75.0-75.0) | 263.4 (263.4-263.4) |
| 2009 | 0.1 (0.1-0.1) | 1.1 (1.1-1.1) | 11.0 (11.0-11.0) | 74.0 (74.0-74.0) | 257.5 (257.5-257.5) |
| 2010 | 0.1 (0.1-0.1) | 1.1 (1.1-1.1) | 11.0 (11.0-11.0) | 78.3 (78.3-78.3) | 278.3 (278.3-278.3) |
| 2011 | 0.1 (0.1-0.1) | 1.3 (1.3-1.3) | 11.8 (11.8-11.8) | 79.9 (79.9-79.9) | 283.3 (283.3-283.3) |
| 2012 | 0.1 (0.1-0.1) | 1.4 (1.4-1.4) | 11.4 (11.4-11.4) | 82.1 (82.1-82.1) | 290.6 (290.6-290.6) |
| 2013 | 0.1 (0.1-0.1) | 1.3 (1.3-1.3) | 11.2 (11.2-11.2) | 81.7 (81.7-81.7) | 290.6 (290.6-290.6) |
| 2014 | 0.1 (0.1-0.1) | 1.1 (1.1-1.1) | 11.3 (11.3-11.3) | 78.0 (78.0-78.0) | 279.8 (279.8-279.8) |
| 2015 | 0.1 (0.1-0.1) | 1.2 (1.2-1.2) | 11.4 (11.4-11.4) | 77.9 (77.9-77.9) | 280.1 (280.1-280.1) |
| 2016 | 0.1 (0.1-0.1) | 1.2 (1.2-1.2) | 11.8 (11.8-11.8) | 76.5 (76.5-76.5) | 283.3 (283.3-283.3) |
| 2017 | 0.1 (0.1-0.1) | 1.3 (1.3-1.3) | 12.4 (12.4-12.4) | 78.1 (78.1-78.1) | 288.5 (288.5-288.5) |
| 2018 | 0.1 (0.1-0.1) | 1.5 (1.5-1.5) | 12.1 (12.1-12.1) | 79.9 (79.9-79.9) | 289.1 (289.1-289.1) |
| 2019 | 0.1 (0.1-0.1) | 1.4 (1.4-1.4) | 12.5 (12.5-12.5) | 80.2 (80.2-80.2) | 290.7 (290.7-290.7) |
| 2020 | 0.2 (0.2-0.2) | 1.9 (1.9-1.9) | 18.0 (18.0-18.0) | 111.2 (111.2-111.2) | 396.8 (396.8-396.8) |
| 2021 | 0.2 (0.2-0.2) | 1.9 (1.9-1.9) | 16.8 (16.8-16.8) | 103.1 (103.1-103.1) | 387.4 (387.4-387.4) |
| 2022 | 0.2 (0.2-0.2) | 2.0 (2.0-2.0) | 16.5 (16.5-16.5) | 100.2 (100.2-100.2) | 361.9 (361.9-361.9) |
| 2023 | 0.1 (0.1-0.1) | 1.9 (1.9-1.9) | 15.3 (15.3-15.3) | 92.3 (92.3-92.3) | 351.9 (351.9-351.9) |
| Total | 0.1 (0.1-0.1) | 1.3 (1.2-1.4) | 11.9 (10.9-12.9) | 76.8 (71.0-82.6) | 272.5 (248.9-296.1) |

AAMR: age-adjusted mortality rate; CI: confidence interval

**Supplementary Table 4.** DM and Dementia-related mortality per 100,000 stratified by state group of death in the United States from 1999 to 2023.

| **State** | **AAMR (95% CI)** |
| --- | --- |
| Vermont | 38.8 (35.2-42.3) |
| Oklahoma | 37.8 (31.7-43.9) |
| Oregon | 36.9 (32.2-41.5) |
| Mississippi | 36.4 (31.3-41.5) |
| South Carolina | 36.3 (32.0-40.6) |
| Minnesota | 36.1 (31.1-41.1) |
| West Virginia | 34.5 (31.9-37.0) |
| Kentucky | 34.0 (28.3-39.6) |
| Nebraska | 33.4 (28.6-38.2) |
| Tennessee | 33.1 (30.2-36.1) |
| Ohio | 32.9 (31.0-34.8) |
| North Carolina | 32.7 (31.0-34.4) |
| Texas | 31.3 (28.1-34.4) |
| Washington | 31.0 (28.5-33.4) |
| Maryland | 30.0 (27.6-32.4) |
| Indiana | 28.8 (25.5-32.1) |
| Colorado | 28.4 (23.9-32.8) |
| North Dakota | 28.2 (26.2-30.1) |
| Wisconsin | 28.1 (25.5-30.8) |
| Iowa | 27.7 (25.1-30.3) |
| Rhode Island | 27.6 (23.7-31.6) |
| District of Columbia | 27.5 (24.7-30.3) |
| South Dakota | 27.4 (23.5-31.4) |
| Idaho | 27.3 (24.2-30.4) |
| Alaska | 26.5 (23.5-29.6) |
| Maine | 26.0 (24.2-27.7) |
| Delaware | 25.5 (21.2-29.7) |
| New Hampshire | 25.4 (23.7-27.1) |
| Montana | 24.9 (22.9-26.8) |
| California | 24.9 (23.1-26.7) |
| Alabama | 24.2 (22.4-26.1) |
| Wyoming | 23.4 (19.4-27.4) |
| Michigan | 23.0 (21.7-24.2) |
| Virginia | 23.0 (20.9-25.1) |
| Georgia | 22.7 (21.2-24.3) |
| Pennsylvania | 22.4 (21.0-23.8) |
| Arkansas | 22.3 (19.4-25.1) |
| Utah | 22.0 (19.8-24.2) |
| Hawaii | 21.9 (20.2-23.5) |
| Missouri | 21.8 (20.3-23.4) |
| New Mexico | 21.8 (19.9-23.8) |
| Kansas | 21.6 (19.6-23.6) |
| Louisiana | 21.6 (18.2-25.0) |
| Illinois | 19.7 (18.4-21.0) |
| Massachusetts | 18.1 (16.9-19.2) |
| Connecticut | 17.5 (16.5-18.6) |
| New York | 17.4 (15.6-19.2) |
| New Jersey | 16.7 (15.1-18.2) |
| Arizona | 14.3 (12.3-16.3) |
| Florida | 14.3 (12.6-16.0) |
| Nevada | 12.4 (10.1-14.7) |

AAMR: age-adjusted mortality rate; CI: confidence interval

**Supplementary Table 5.** DM and Dementia-related mortality per 100,000 stratified by urbanization of death in the United States from 1999 to 2020.

| Age-Adjusted Rate /100,000 (95% CI) | | |
| --- | --- | --- |
| Year | **Metropolitan** | **Nonmetropolitan** |
| 1999 | 11.7 (11.4-11.9) | 13.7 (13.2-14.2) |
| 2000 | 17.3 (17.0-17.6) | 19.5 (18.9-20.2) |
| 2001 | 18.4 (18.1-18.7) | 20.3 (19.7-21.0) |
| 2002 | 19.5 (19.2-19.9) | 22.9 (22.2-23.6) |
| 2003 | 20.4 (20.1-20.7) | 24.2 (23.5-24.9) |
| 2004 | 20.6 (20.3-20.9) | 24.4 (23.7-25.1) |
| 2005 | 21.9 (21.6-22.2) | 26.4 (25.6-27.1) |
| 2006 | 21.9 (21.6-22.2) | 25.9 (25.2-26.6) |
| 2007 | 22.5 (22.2-22.8) | 27.3 (26.6-28.0) |
| 2008 | 22.9 (22.5-23.2) | 28.2 (27.5-29.0) |
| 2009 | 22.2 (21.9-22.5) | 29.0 (28.2-29.7) |
| 2010 | 23.8 (23.4-24.1) | 29.9 (29.2-30.7) |
| 2011 | 24.5 (24.2-24.8) | 30.2 (29.5-31.0) |
| 2012 | 25.0 (24.7-25.3) | 31.0 (30.3-31.8) |
| 2013 | 25.0 (24.7-25.3) | 30.2 (29.5-30.9) |
| 2014 | 24.0 (23.7-24.3) | 29.4 (28.6-30.1) |
| 2015 | 23.9 (23.6-24.2) | 30.0 (29.3-30.7) |
| 2016 | 24.0 (23.7-24.3) | 30.1 (29.4-30.8) |
| 2017 | 24.3 (24.0-24.6) | 32.0 (31.2-32.7) |
| 2018 | 24.6 (24.3-24.9) | 32.2 (31.4-32.9) |
| 2019 | 24.5 (24.2-24.8) | 33.4 (32.6-34.1) |
| 2020 | 34.3 (34.0-34.7) | 43.8 (42.9-44.6) |
| Total | 23.8 (23.4-24.2) | 28.6 (28.0-29.2) |

AAMR: age-adjusted mortality rate; CI: confidence interval

**Supplementary Table 6.** DM and Dementia-related mortality per 100,000 stratified by census region of death in the United States from 1999 to 2023.

| Age-Adjusted Rate /100,000 (95% CI) | | | | |
| --- | --- | --- | --- | --- |
| Year | **Northeast** | **Midwest** | **South** | **West** |
| 1999 | 10.4 (10.0-10.9) | 12.9 (12.5-13.4) | 13.4 (13.0-13.8) | 10.3 (9.9-10.8) |
| 2000 | 15.2 (14.7-15.7) | 19.8 (19.2-20.4) | 18.0 (17.5-18.4) | 17.5 (16.9-18.1) |
| 2001 | 15.8 (15.2-16.3) | 20.7 (20.2-21.3) | 19.0 (18.5-19.4) | 19.6 (18.9-20.2) |
| 2002 | 16.7 (16.2-17.3) | 22.4 (21.8-23.0) | 20.7 (20.2-21.2) | 20.4 (19.7-21.0) |
| 2003 | 16.4 (15.8-16.9) | 23.7 (23.1-24.3) | 22.3 (21.8-22.8) | 20.9 (20.3-21.6) |
| 2004 | 17.3 (16.7-17.8) | 24.1 (23.5-24.7) | 21.8 (21.4-22.3) | 21.5 (20.9-22.2) |
| 2005 | 18.0 (17.4-18.5) | 25.7 (25.0-26.3) | 23.6 (23.1-24.1) | 22.8 (22.2-23.5) |
| 2006 | 17.8 (17.3-18.4) | 25.5 (24.8-26.1) | 23.2 (22.7-23.7) | 23.1 (22.4-23.7) |
| 2007 | 18.4 (17.9-19.0) | 26.3 (25.7-26.9) | 24.5 (23.9-25.0) | 23.3 (22.6-23.9) |
| 2008 | 18.4 (17.8-19.0) | 26.8 (26.1-27.4) | 25.1 (24.6-25.6) | 23.9 (23.3-24.6) |
| 2009 | 18.2 (17.6-18.7) | 26.3 (25.7-27.0) | 24.7 (24.2-25.2) | 22.9 (22.3-23.5) |
| 2010 | 19.4 (18.9-20.0) | 26.8 (26.2-27.4) | 26.6 (26.1-27.1) | 25.1 (24.5-25.7) |
| 2011 | 20.5 (19.9-21.0) | 28.0 (27.3-28.6) | 26.5 (26.0-27.0) | 25.9 (25.2-26.5) |
| 2012 | 20.5 (19.9-21.1) | 28.5 (27.9-29.2) | 27.4 (26.9-27.9) | 26.2 (25.6-26.8) |
| 2013 | 20.4 (19.9-21.0) | 27.8 (27.2-28.4) | 27.1 (26.6-27.6) | 27.1 (26.4-27.7) |
| 2014 | 20.2 (19.6-20.7) | 27.9 (27.3-28.6) | 25.5 (25.1-26.0) | 25.2 (24.6-25.8) |
| 2015 | 20.1 (19.5-20.6) | 27.8 (27.2-28.4) | 25.3 (24.8-25.7) | 25.8 (25.2-26.4) |
| 2016 | 19.7 (19.1-20.2) | 27.1 (26.5-27.7) | 25.6 (25.1-26.0) | 26.6 (26.0-27.2) |
| 2017 | 20.1 (19.6-20.7) | 28.2 (27.6-28.8) | 26.1 (25.7-26.6) | 26.9 (26.3-27.5) |
| 2018 | 21.0 (20.4-21.5) | 27.7 (27.1-28.3) | 27.4 (27.0-27.9) | 25.6 (25.0-26.1) |
| 2019 | 20.8 (20.3-21.4) | 27.8 (27.2-28.4) | 27.5 (27.1-28.0) | 26.0 (25.5-26.6) |
| 2020 | 29.8 (29.2-30.5) | 38.8 (38.1-39.5) | 38.5 (37.9-39.0) | 34.0 (33.3-34.6) |
| 2021 | 25.1 (24.5-25.7) | 34.3 (33.6-35.0) | 38.4 (37.9-38.9) | 35.0 (34.3-35.7) |
| 2022 | 24.1 (23.5-24.6) | 31.6 (31.0-32.2) | 36.8 (36.3-37.3) | 34.1 (33.5-34.8) |
| 2023 | 22.5 (21.9-23.0) | 30.5 (29.8-31.1) | 35.8 (35.3-36.3) | 30.4 (29.8-31.0) |
| Total | 20.6 (19.8 –21.4) | 26.9 (26.1-27.7) | 26.8 (26.2-27.4) | 25.4 (24.7-26.1) |

AAMR: age-adjusted mortality rate; CI: confidence interval


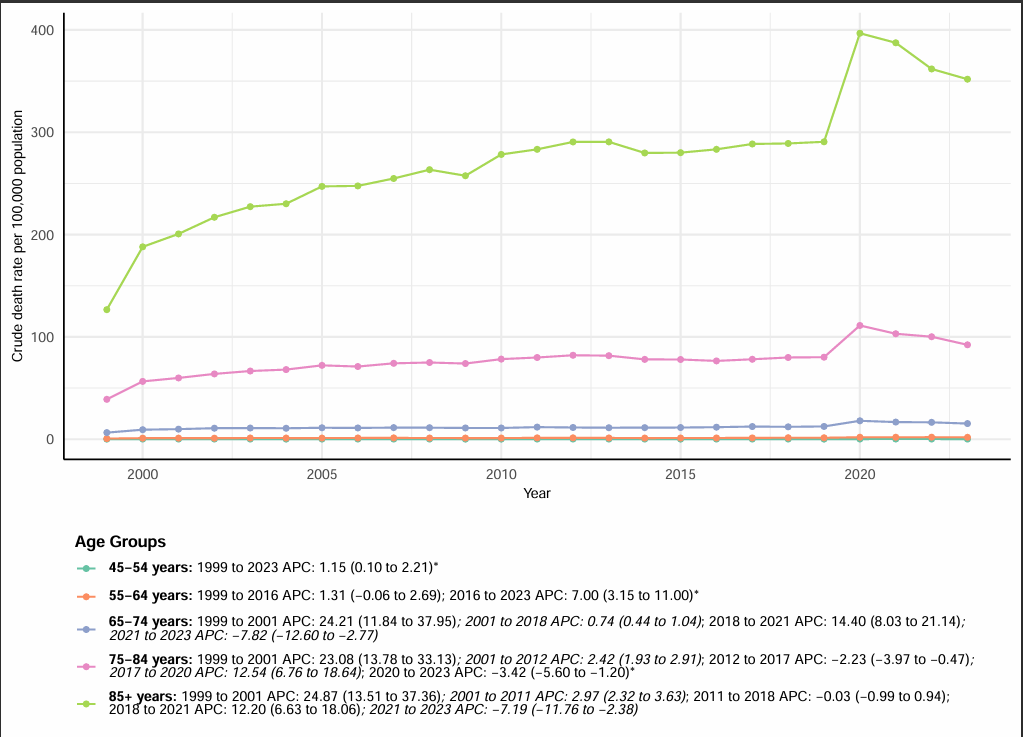


**Supplementary Figure 3.** Trends in DM and Dementia-related crude mortality rates stratified by the age groups in the United States, 1999 to 2023.

APC = Annual Percentage Change, CI = Confidence Interval.

*Indicates that the Annual Percentage Change (APC) is significantly different from zero at α = 0.05.

| **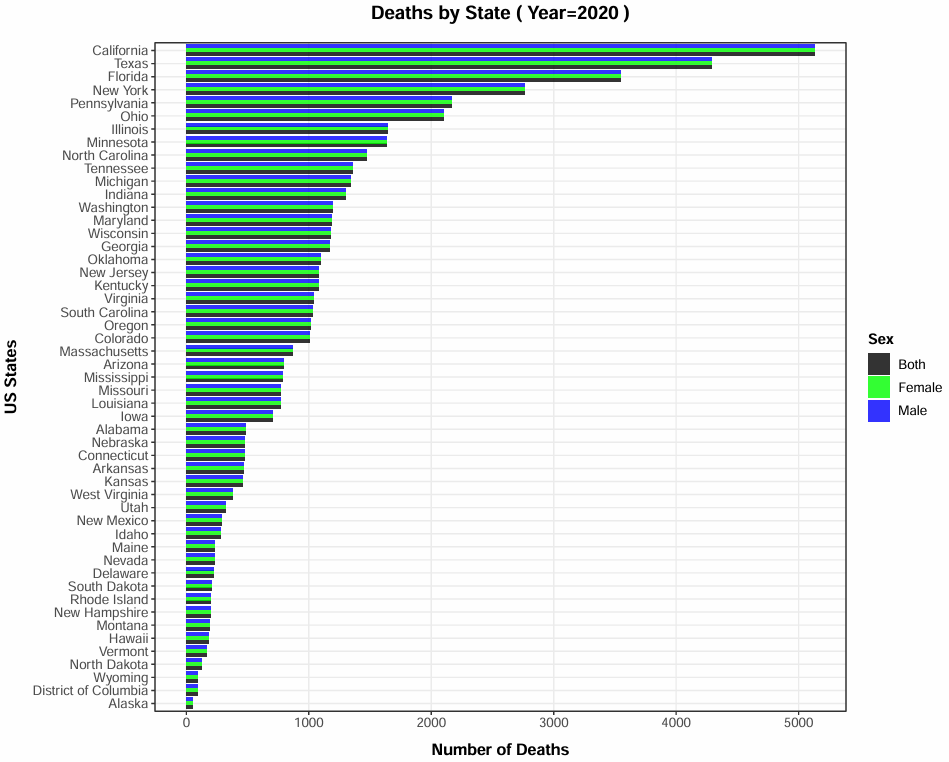**  A | **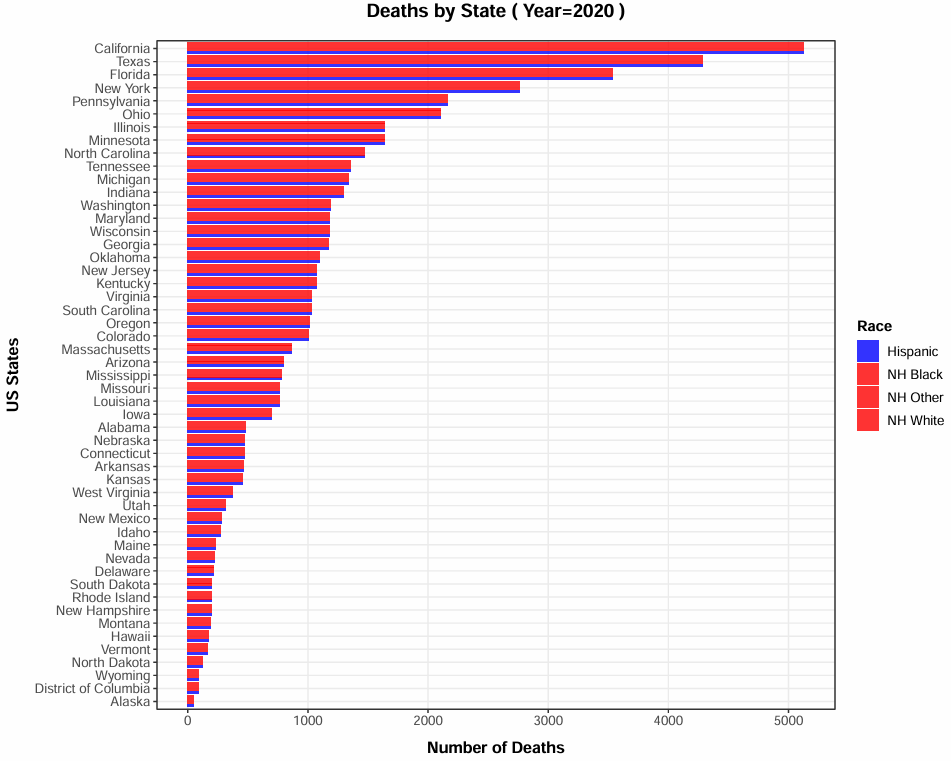**  B |
| --- | --- |

**Supplementary Figure 4.** State-level ranking of deaths from dementia associated with diabetes, by sex and race/ethnicity, United States, 2020.

A shows rankings by sex (male, female, and both sexes), and B shows rankings by race/ethnicity (NH White, NH Black, Hispanic, and NH Other). ars represent the total number of deaths in each state. NH = non-Hispanic.


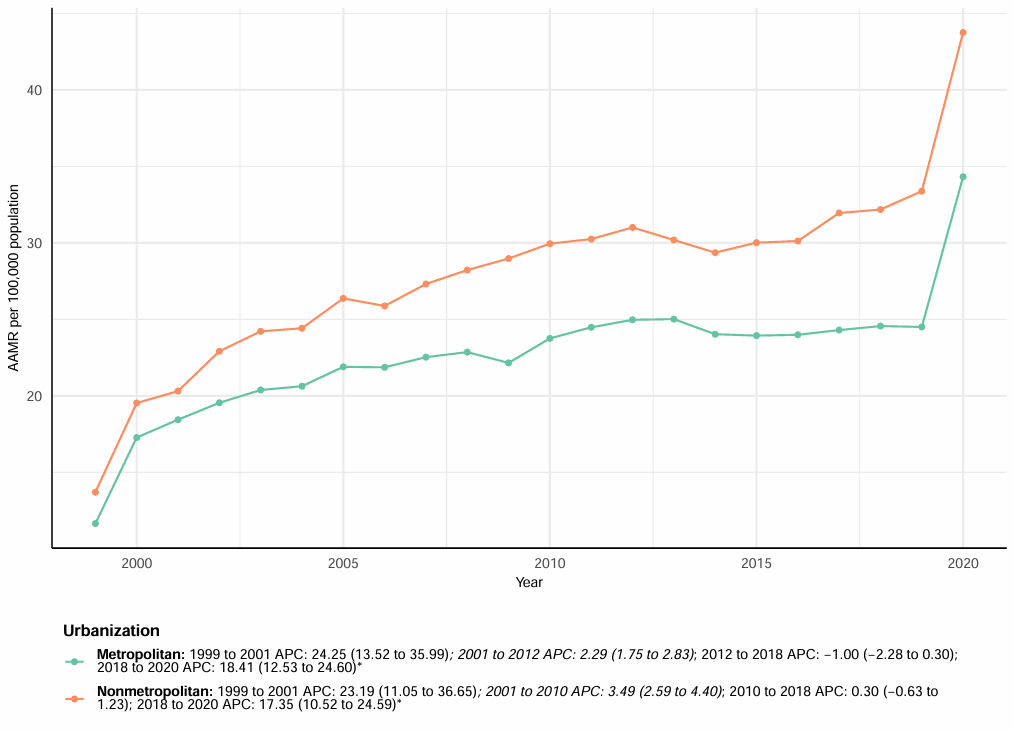


**Supplementary Figure 5.** Trends in Diabetes and Dementia-related mortality stratified by the level of urbanisation in the United States from 1999 to 2020.

APC = Annual Percentage Change, CI = Confidence Interval.

*Indicates that the Annual Percentage Change (APC) is significantly different from zero at α = 0.05.


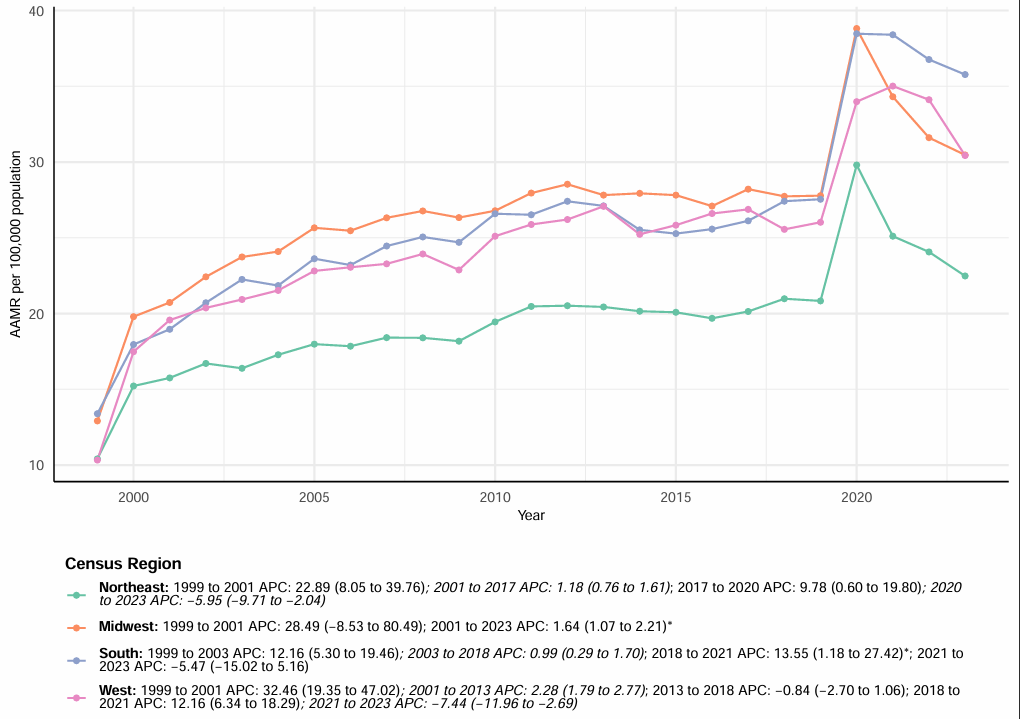


**Supplementary Figure 6.** Trends in Diabetes and Dementia-related mortality stratified by the Census Region in the United States from 1999 to 2023.

APC = Annual Percentage Change, CI = Confidence Interval.

*Indicates that the Annual Percentage Change (APC) is significantly different from zero at α = 0.05.

**Supplementary Table 7.** Parallelism tests for subgroup-specific mortality trends using Joinpoint regression.

| Subgroup variable | Groups compared | Numerator  *df* | Denominator  *df* | Number of permutations | *P*-value | Interpretation |
| --- | --- | --- | --- | --- | --- | --- |
| Sex | Male vs Female | 9 | 30 | 4500 | 0.022 | Not parallel |
| Race | Hispanic vs NH Black | 9 | 30 | 4500 | <0.001 | Not parallel |
|  | Hispanic vs NH Other | 9 | 30 | 4500 | 0.148 | Parallel |
|  | Hispanic vs NH White | 9 | 30 | 4500 | 0.129 | Parallel |
|  | NH Black vs NH Other | 7 | 34 | 4500 | 0.406 | Parallel |
|  | NH Black vs NH White | 9 | 30 | 4500 | 0.020 | Not parallel |
|  | NH Other vs NH White | 9 | 30 | 4500 | 0.903 | Parallel |
| Census Region | Midwest vs Northeast | 9 | 30 | 4500 | 0.003 | Not parallel |
|  | Midwest vs South | 9 | 30 | 4500 | 0.007 | Not parallel |
|  | Midwest vs West | 9 | 30 | 4500 | <0.001 | Not parallel |
|  | Northeast vs South | 9 | 30 | 4500 | 0.045 | Not parallel |
|  | Northeast vs West | 9 | 30 | 4500 | 0.002 | Not parallel |
|  | South vs West | 9 | 30 | 4500 | 0.06 | Parallel |
| Age group | 45-54 vs 55-64 | 7 | 34 | 4500 | 0.104 | Parallel |
|  | 45-54 vs 65-74 | 7 | 34 | 4500 | 0.951 | Parallel |
|  | 45-54 vs 75-84 | 9 | 30 | 4500 | 0.981 | Parallel |
|  | 45-54 vs 85+ | 9 | 30 | 4500 | 0.980 | Parallel |
|  | 55-64 vs 65-74 | 7 | 34 | 4500 | 0.814 | Parallel |
|  | 55-64 vs 75-84 | 9 | 30 | 4500 | 0.732 | Parallel |
|  | 55-64 vs 85+ | 9 | 30 | 4500 | 0.810 | Parallel |
|  | 65-74 vs 75-84 | 9 | 30 | 4500 | 0.070 | Parallel |
|  | 65-74 vs 84+ | 9 | 30 | 4500 | 0.062 | Parallel |
|  | 75-84 vs 85+ | 9 | 30 | 4500 | 0.059 | Parallel |
| Urbanization^#^ | Metropolitan vs  Non-metropolitan | 7 | 28 | 4500 | <0.001 | Not parallel |

Note: ^#^Urbanization data were available only for 1999-2020.

**Supplementary Table 8.** Trends in deaths and age-adjusted mortality rates (AAMR) for DM and dementia in the United States, 1999-2019.

| **Characteristic** | **Deaths** | | | | |  | **AAMR** | | |
| --- | --- | --- | --- | --- | --- | --- | --- | --- | --- |
|  | **Overall** | **Deaths_1999** | **Deaths_2019** | | **Percent change** |  | **AAMR_1999** | **AAMR_2019** | **AAPC (95% CI)** |
| **Both** | 559284 | 11382 | 36573 | 221.32 | |  | 12.05 (11.83 to 12.28) | 26.02 (25.75 to 26.28) | 3.48 (2.57 to 4.39*) |
| **Sex** |  |  |  |  | |  |  |  |  |
| Female | 339525 | 7247 | 20786 | 186.82 | |  | 11.79 (11.52 to 12.07) | 24.58 (24.24 to 24.92) | 3.30 (2.37 to 4.23*) |
| Male | 219759 | 4135 | 15787 | 281.79 | |  | 12.26 (11.88 to 12.64) | 27.91 (27.48 to 28.35) | 3.74 (2.78 to 4.70*) |
| **Census Region** |  |  |  |  | |  |  |  |  |
| Northeast | 92269 | 2133 | 5687 | 166.62 | |  | 10.41 (9.97 to 10.85) | 20.83 (20.29 to 21.38) | 3.26 (1.99 to 4.55*) |
| Midwest | 142625 | 2992 | 8527 | 184.99 | |  | 12.91 (12.45 to 13.38) | 27.78 (27.19 to 28.37) | 3.49 (2.53 to 4.46*) |
| South | 205537 | 4368 | 14279 | 226.90 | |  | 13.39 (12.99 to 13.79) | 27.54 (27.09 to 28.00) | 3.48 (2.27 to 4.71*) |
| West | 118853 | 1889 | 8080 | 327.74 | |  | 10.33 (9.86 to 10.79) | 26.02 (25.45 to 26.60) | 4.15 (2.81 to 5.50*) |
| **Race** |  |  |  |  | |  |  |  |  |
| Hispanic | 39164 | 433 | 3401 | 685.45 | |  | 11.76 (10.64 to 12.89) | 29.95 (28.93 to 30.96) | 4.49 (3.12 to 5.88*) |
| NH Black | 69178 | 1392 | 4425 | 217.89 | |  | 19.29 (18.28 to 20.31) | 36.82 (35.72 to 37.91) | 2.56 (1.42 to 3.72*) |
| NH White | 433132 | 9359 | 27235 | 191.00 | |  | 11.47 (11.24 to 11.71) | 24.76 (24.47 to 25.06) | 3.46 (2.60 to 4.32*) |
| NH Other | 16650 | 169 | 1447 | 756.21 | |  | 8.57 (7.24 to 9.89) | 19.84 (18.81 to 20.87) | 2.38 (1.24 to 3.53*) |
| **Urbanization^#^** |  |  |  |  | |  |  |  |  |
| Metropolitan | 440682 | 8856 | 28692 | 223.98 | |  | 11.65 (11.41 to 11.90) | 24.50 (24.22 to 24.79) | 3.34 (2.45 to 4.23*) |
| Nonmetropolitan | 118602 | 2526 | 7881 | 212.00 | |  | 13.70 (13.17 to 14.24) | 33.38 (32.64 to 34.12) | 4.05 (2.82 to 5.29*) |
| **Age Group^##^** |  |  |  |  | |  |  |  |  |
| 45-54 years | 1054 | 25 | 43 | 72.00 | |  | 0.07 (0.07 to 0.07) | 0.11 (0.11 to 0.11) | 0.12 (-1.15 to 1.41) |
| 55-64 years | 8719 | 143 | 594 | 315.38 | |  | 0.60 (0.60 to 0.60) | 1.40 (1.40 to 1.40) | 1.55 (0.73 to 2.38*) |
| 65-74 years | 53242 | 1187 | 3934 | 231.42 | |  | 6.44 (6.44 to 6.44) | 12.50 (12.50 to 12.50) | 2.90 (1.88 to 3.94*) |
| 75-84 years | 204389 | 4766 | 12803 | 168.63 | |  | 38.99 (38.99 to 38.99) | 80.17 (80.17 to 80.17) | 3.21 (2.24 to 4.19*) |
| 85+ years | 291880 | 5261 | 19199 | 264.93 | |  | 126.65 (126.65 to 126.65) | 290.68 (290.68 to 290.68) | 3.81 (2.88 to 4.75*) |

Note: **P*<0.05; ^#^ Urbanization data were available only for 1999-2020. For this subgroup, values reported in the columns “Deaths_2019” and “AAMR_2019” correspond to estimates from 2020. Percent change and AAPC for the urbanization subgroup were calculated for the period 1999-2020 only; ^##^AAMRs for age groups were derived from crude rates; NH = non-Hispanic; AAMR = age-adjusted mortality rate per 100,000 population; AAPC=average annual percent change.

**Supplementary Table 9.** Excess mortality during the COVID-19 pandemic (2020-2023).

| **Year** | **Characteristic** | **Observed AAMR** | | **Expected AAMR** | | **Excess AAMR** | | **Excess (%)** |
| --- | --- | --- | --- | --- | --- | --- | --- | --- |
| **2020** |  |  |  | |  | |  | |
|  | **Both** | 35.93 (35.62 to 36.24) | 29.28 (26.33 to 32.56) | | 6.65 | | 22.71 | |
|  | **Sex** |  |  | |  | |  | |
|  | Female | 33.95 (33.55 to 34.34) | 27.89 (24.92 to 31.21) | | 6.06 | | 21.71 | |
|  | Male | 38.51 (38.00 to 39.02) | 31.18 (28.22 to 34.46) | | 7.33 | | 23.50 | |
|  | **Census Region** |  |  | |  | |  | |
|  | Midwest | 38.81 (38.11 to 39.51) | 31.83 (28.31 to 35.80) | | 6.98 | | 21.93 | |
|  | Northeast | 29.81 (29.15 to 30.46) | 22.85 (20.90 to 24.99) | | 6.95 | | 30.42 | |
|  | South | 38.47 (37.94 to 39.00) | 30.40 (27.55 to 33.55) | | 8.07 | | 26.53 | |
|  | West | 33.99 (33.34 to 34.63) | 30.46 (26.61 to 34.85) | | 3.53 | | 11.59 | |
|  | **Race** |  |  | |  | |  | |
|  | Hispanic | 46.22 (44.99 to 47.45) | 35.81 (31.44 to 40.78) | | 10.41 | | 29.08 | |
|  | NH Black | 56.63 (55.29 to 57.97) | 42.12 (36.74 to 48.29) | | 14.51 | | 34.44 | |
|  | NH White | 32.90 (32.56 to 33.24) | 27.60 (24.94 to 30.54) | | 5.30 | | 19.22 | |
|  | NH Other | 28.69 (27.49 to 29.89) | 24.36 (21.20 to 27.99) | | 4.33 | | 17.78 | |
|  | **Age Groups^##^** |  |  | |  | |  | |
|  | 45-54 years | 0.16 (0.16 to 0.16) | 0.12 (0.10 to 0.15) | | 0.04 | | 29.31 | |
|  | 55-64 years | 1.94 (1.94 to 1.94) | 1.44 (1.27 to 1.64) | | 0.50 | | 34.52 | |
|  | 65-74 years | 18.00 (18.00 to 18.00) | 12.97 (11.80 to 14.25) | | 5.03 | | 38.79 | |
|  | 75-84 years | 111.16 (111.16 to 111.16) | 90.01 (80.97 to 100.06) | | 21.16 | | 23.51 | |
|  | 85+ years | 396.78 (396.78 to 396.78) | 333.82 (298.83 to 372.92) | | 62.95 | | 18.86 | |
| **2021** |  |  |  | |  | |  | |
|  | **Both** | 34.24 (33.93 to 34.56) | 29.98 (26.76 to 33.58) | | 4.27 | | 14.24 | |
|  | **Sex** |  |  | |  | |  | |
|  | Female | 32.63 (32.23 to 33.03) | 28.49 (25.25 to 32.13) | | 4.14 | | 14.52 | |
|  | Male | 36.43 (35.92 to 36.94) | 32.01 (28.77 to 35.63) | | 4.42 | | 13.79 | |
|  | **Census Region** |  |  | |  | |  | |
|  | Midwest | 34.31 (33.63 to 34.99) | 32.54 (28.70 to 36.90) | | 1.77 | | 5.43 | |
|  | Northeast | 25.11 (24.50 to 25.72) | 23.34 (21.20 to 25.68) | | 1.77 | | 7.59 | |
|  | South | 38.40 (37.86 to 38.95) | 31.12 (28.00 to 34.58) | | 7.29 | | 23.41 | |
|  | West | 35.01 (34.34 to 35.69) | 31.27 (27.07 to 36.13) | | 3.74 | | 11.95 | |
|  | **Race** |  |  | |  | |  | |
|  | Hispanic | 39.32 (38.17 to 40.46) | 36.98 (32.17 to 42.51) | | 2.34 | | 6.32 | |
|  | NH Black | 50.61 (49.30 to 51.91) | 42.72 (36.91 to 49.45) | | 7.88 | | 18.45 | |
|  | NH White | 32.38 (32.03 to 32.73) | 28.25 (25.35 to 31.49) | | 4.12 | | 14.60 | |
|  | NH Other | 26.43 (25.33 to 27.53) | 25.04 (21.58 to 29.06) | | 1.38 | | 5.52 | |
|  | **Age Groups^##^** |  |  | |  | |  | |
|  | 45-54 years | 0.17 (0.17 to 0.17) | 0.12 (0.10 to 0.15) | | 0.05 | | 39.70 | |
|  | 55-64 years | 1.92 (1.92 to 1.92) | 1.47 (1.28 to 1.69) | | 0.45 | | 30.54 | |
|  | 65-74 years | 16.76 (16.76 to 16.76) | 13.17 (11.91 to 14.58) | | 3.58 | | 27.20 | |
|  | 75-84 years | 103.07 (103.07 to 103.07) | 91.95 (82.10 to 102.98) | | 11.12 | | 12.09 | |
|  | 85+ years | 387.45 (387.45 to 387.45) | 342.86 (304.54 to 386.02) | | 44.58 | | 13.00 | |
| **2022** |  |  |  | |  | |  | |
|  | **Both** | 32.70 (32.41 to 33.00) | 30.69 (27.18 to 34.64) | | 2.02 | | 6.58 | |
|  | **Sex** |  |  | |  | |  | |
|  | Female | 30.75 (30.38 to 31.12) | 29.10 (25.59 to 33.10) | | 1.64 | | 5.64 | |
|  | Male | 35.41 (34.92 to 35.89) | 32.87 (29.33 to 36.84) | | 2.54 | | 7.71 | |
|  | **Census Region** |  |  | |  | |  | |
|  | Midwest | 31.61 (30.98 to 32.24) | 33.27 (29.09 to 38.04) | | -1.66 | | -4.98 | |
|  | Northeast | 24.07 (23.49 to 24.65) | 23.83 (21.51 to 26.40) | | 0.24 | | 0.99 | |
|  | South | 36.77 (36.26 to 37.28) | 31.85 (28.46 to 35.64) | | 4.92 | | 15.44 | |
|  | West | 34.11 (33.48 to 34.75) | 32.12 (27.53 to 37.47) | | 2.00 | | 6.22 | |
|  | **Race** |  |  | |  | |  | |
|  | Hispanic | 39.25 (38.14 to 40.35) | 38.19 (32.91 to 44.31) | | 1.06 | | 2.77 | |
|  | NH Black | 47.56 (46.33 to 48.79) | 43.33 (37.07 to 50.65) | | 4.23 | | 9.75 | |
|  | NH White | 30.89 (30.56 to 31.21) | 28.93 (25.77 to 32.48) | | 1.96 | | 6.77 | |
|  | NH Other | 24.63 (23.63 to 25.64) | 25.75 (21.97 to 30.18) | | -1.12 | | -4.33 | |
|  | **Age Groups^##^** |  |  | |  | |  | |
|  | 45-54 years | 0.18 (0.18 to 0.18) | 0.12 (0.10 to 0.15) | | 0.06 | | 47.98 | |
|  | 55-64 years | 2.00 (2.00 to 2.00) | 1.50 (1.30 to 1.74) | | 0.50 | | 33.05 | |
|  | 65-74 years | 16.49 (16.49 to 16.49) | 13.38 (12.01 to 14.91) | | 3.11 | | 23.23 | |
|  | 75-84 years | 100.17 (100.17 to 100.17) | 93.94 (83.24 to 106.01) | | 6.24 | | 6.64 | |
|  | 85+ years | 361.86 (361.86 to 361.86) | 352.15 (310.31 to 399.63) | | 9.71 | | 2.76 | |
| **2023** |  |  |  | |  | |  | |
|  | **Both** | 30.95 (30.66 to 31.24) | 31.41 (27.61 to 35.73) | | -0.46 | | -1.47 | |
|  | **Sex** |  |  | |  | |  | |
|  | Female | 29.24 (28.88 to 29.61) | 29.73 (25.93 to 34.09) | | -0.49 | | -1.64 | |
|  | Male | 33.15 (32.68 to 33.62) | 33.75 (29.89 to 38.10) | | -0.60 | | -1.77 | |
|  | Census Region |  |  | |  | |  | |
|  | Midwest | 30.47 (29.85 to 31.09) | 34.01 (29.49 to 39.22) | | -3.54 | | -10.41 | |
|  | Northeast | 22.48 (21.92 to 23.04) | 24.33 (21.83 to 27.13) | | -1.85 | | -7.62 | |
|  | South | 35.77 (35.27 to 36.28) | 32.60 (28.92 to 36.74) | | 3.18 | | 9.75 | |
|  | West | 30.43 (29.83 to 31.03) | 32.98 (28.00 to 38.85) | | -2.55 | | -7.74 | |
|  | **Race** |  |  | |  | |  | |
|  |  |  |  | |  | |  | |
|  | Hispanic | 34.52 (33.49 to 35.55) | 39.44 (33.67 to 46.19) | | -4.91 | | -12.46 | |
|  | NH Black | 45.15 (43.96 to 46.34) | 43.95 (37.23 to 51.89) | | 1.20 | | 2.73 | |
|  | NH White | 29.55 (29.22 to 29.87) | 29.62 (26.19 to 33.50) | | -0.07 | | -0.25 | |
|  | NH Other | 22.76 (21.81 to 23.71) | 26.47 (22.36 to 31.34) | | -3.72 | | -14.04 | |
|  | **Age Groups^##^** |  |  | |  | |  | |
|  | 45-54 years | 0.13 (0.13 to 0.13) | 0.12 (0.10 to 0.15) | | 0.00 | | 1.39 | |
|  | 55-64 years | 1.85 (1.85 to 1.85) | 1.53 (1.31 to 1.79) | | 0.32 | | 21.06 | |
|  | 65-74 years | 15.31 (15.31 to 15.31) | 13.60 (12.12 to 15.25) | | 1.71 | | 12.59 | |
|  | 75-84 years | 92.27 (92.27 to 92.27) | 95.97 (84.39 to 109.13) | | -3.70 | | -3.85 | |
|  | 85+ years | 351.87 (351.87 to 351.87) | 361.69 (316.16 to 413.77) | | -9.82 | | -2.72 | |

Note: ^##^AAMRs for age groups were derived from crude rates; NH = non-Hispanic; AAMR = age-adjusted mortality rate per 100,000 population.

**Supplementary Table 10.** Deaths and Percentage of Total Deaths (%) According to Underlying and Contributing Causes of Death (DM and Dementia).

| **Year** | **DM as Underlying Cause of Death** | | **Dementia as Underlying Cause of Death** | |
| --- | --- | --- | --- | --- |
|  | **Deaths** | **Percent of Total Deaths (%)** | **Deaths** | **Percent of Total Deaths (%)** |
| 1999 | 3096 | 2.35 | 3019 | 1.04 |
| 2000 | 4704 | 3.58 | 3530 | 1.21 |
| 2001 | 5068 | 3.85 | 4069 | 1.40 |
| 2002 | 5437 | 4.13 | 4557 | 1.56 |
| 2003 | 5643 | 4.29 | 5137 | 1.76 |
| 2004 | 5772 | 4.39 | 5413 | 1.86 |
| 2005 | 6126 | 4.66 | 6560 | 2.26 |
| 2006 | 4606 | 3.50 | 9104 | 3.13 |
| 2007 | 4818 | 3.66 | 9423 | 3.24 |
| 2008 | 4774 | 3.63 | 11072 | 3.81 |
| 2009 | 4588 | 3.49 | 11118 | 3.82 |
| 2010 | 4968 | 3.78 | 12502 | 4.30 |
| 2011 | 5224 | 3.97 | 13557 | 4.66 |
| 2012 | 5400 | 4.11 | 14350 | 4.93 |
| 2013 | 5237 | 3.98 | 14731 | 5.07 |
| 2014 | 5215 | 3.97 | 14382 | 4.95 |
| 2015 | 5235 | 3.98 | 14431 | 4.96 |
| 2016 | 5280 | 4.02 | 14602 | 5.02 |
| 2017 | 5448 | 4.14 | 15288 | 5.26 |
| 2018 | 5404 | 4.11 | 15573 | 5.35 |
| 2019 | 5327 | 4.05 | 15955 | 5.49 |
| 2020 | 6466 | 4.92 | 18948 | 6.52 |
| 2021 | 6029 | 4.58 | 17766 | 6.11 |
| 2022 | 5924 | 4.50 | 18261 | 6.28 |
| 2023 | 5739 | 4.36 | 17483 | 6.01 |
| **Total** | 131528 |  | 290831 | 100 |

Note: DM: Diabetes Mellitus; The table presents two mutually exclusive categories:

(1) DM as the underlying cause with dementia listed as a contributing cause, and

(2) dementia as the underlying cause with DM listed as a contributing cause.

**Supplementary Table 11.** External validation of ARIMA forecasts using pre-pandemic data (1999-2017).

| **Outcome** | **Sex** | **Year** | **Actual (95% CI)** | **Predicted (95% CI)^#^** | **RMSE** | **MAE** | **MAPE(%)** |
| --- | --- | --- | --- | --- | --- | --- | --- |
| AAMR | Male | 2018 | 27.72 (27.28 to 28.16) | 28.05 (25.32 to 30.77) | 0.64 | 0.59 | 2.11 |
|  |  | 2019 | 27.91 (27.48 to 28.35) | 28.76 (24.29 to 33.23) |  |  |  |
| AAMR | Female | 2018 | 24.48 (24.14 to 24.82) | 24.36 (21.77 to 26.95) | 0.10 | 0.09 | 0.37 |
|  |  | 2019 | 24.58 (24.24 to 24.92) | 24.64 (20.02 to 29.26) |  |  |  |
| AAMR | Both | 2018 | 25.86 (25.58 to 26.13) | 26.18 (23.44 to 28.92) | 0.57 | 0.53 | 2.05 |
|  |  | 2019 | 26.02 (25.75 to 26.28) | 26.76 (20.62 to 32.89) |  |  |  |
| Deaths | Male | 2018 | 15 227 | 15 156.06 (14 299.90 to 16 012.21) | 61.74 | 60.92 | 0.39 |
|  |  | 2019 | 15 787 | 15 736.11 (14 525.33 to 16 946.90) |  |  |  |
| Deaths | Female | 2018 | 20 353 | 20 268.30 (18 563.13 to 21 973.48) | 68.43 | 65.75 | 0.32 |
|  |  | 2019 | 20 786 | 20 832.80 (17 760.40 to 23 905.20) |  |  |  |
| Deaths | Both | 2018 | 35 580 | 35 711.50 (33 219.06 to 38 203.94) | 310.53 | 275.25 | 0.76 |
|  |  | 2019 | 36 573 | 36 992.00 (33 467.15 to 40 516.85) |  |  |  |

Note: **^#^**Predicted values and 95% confidence intervals (CIs) were obtained from ARIMA models trained on data from 1999-2017. RMSE, MAE, and MAPE represent out-of-sample prediction errors calculated by comparing predicted and observed values in 2018-2019. RMSE: Root Mean Squared Error; MAE: Mean Absolute Error; MAPE (%): Mean Absolute Percentage Error.
